# Healthy Prenatal Dietary Pattern and Offspring Autism

**DOI:** 10.1001/jamanetworkopen.2024.22815

**Published:** 2024-07-18

**Authors:** Catherine Friel, Alastair H. Leyland, Jana J. Anderson, Alexandra Havdahl, Anne Lise Brantsæter, Ruth Dundas

**Affiliations:** 1 MRC/CSO Social and Public Health Sciences Unit, University of Glasgow, Glasgow, Scotland; 2 Public Health Research Group, School of Health & Wellbeing, University of Glasgow, Glasgow, Scotland; 3 Center for Genetic Epidemiology and Mental Health, Norwegian Institute of Public Health, Oslo, Norway; 4 Nic Waals Institute, Lovisenberg Diaconal Hospital, Oslo, Norway; 5 Department of Psychology, Promenta Research Center, University of Oslo, Oslo, Norway; 6 Department of Food Safety, Norwegian Institute of Public Health, Oslo, Norway

## Abstract

**Importance:**

Prenatal diet may be causally related to autism; however, findings are inconsistent, with a limited body of research based on small sample sizes and retrospective study designs.

**Objective:**

To investigate the associations of prenatal dietary patterns with autism diagnosis and autism-associated traits in 2 large prospective cohorts, the Norwegian Mother, Father, and Child Cohort Study (MoBa), and the Avon Longitudinal Study of Parents and Children (ALSPAC).

**Design, Setting, and Participants:**

This cohort study used data from MoBa and ALSPAC birth cohort studies conducted across Norway and in the Southwest of England, respectively. Participants were people with singleton pregnancies with self-reported food frequency questionnaire responses. MoBa recruited between 2002 and 2008, and ALSPAC recruited between 1990 and 1992, and children were followed-up until age 8 years or older. Recruitment rates were 41% (95 200 of 277 702 eligible pregnancies) in MoBa and 72% (14 541 of 20 248 eligible pregnancies) in ALSPAC. Data analysis occurred February 1, 2022, to August 1, 2023.

**Exposure:**

A healthy prenatal dietary pattern was derived using factor analysis and modeled as low, medium, and high adherence.

**Main Outcomes and Measures:**

In MoBa, the offspring outcomes were autism diagnosis and elevated social communication questionnaire score at ages 3 years and 8 years, with further analysis of the social communication difficulties and restrictive and repetitive behaviors subdomains. In ALSPAC, offspring outcomes were elevated social communication difficulties checklist score at age 8 years. Odds ratios (ORs) were estimated using generalized nonlinear models.

**Results:**

MoBa included 84 548 pregnancies (mean [SD] age, 30.2 [4.6] years; 43 277 [51.2%] male offspring) and ALSPAC had 11 760 pregnancies (mean [SD] age, 27.9 [4.7] years; 6034 [51.3%] male offspring). In the final adjusted models, high adherence to a healthy dietary pattern, compared with low adherence, was associated with reduced odds of autism diagnosis (OR, 0.78; 95% CI, 0.66-0.92) and social communication difficulties at age 3 years in MoBa (OR 0.76, 95% CI, 0.70-0.82) and age 8 years in ALSPAC (OR, 0.74; 95% CI, 0.55-0.98). There was no consistent evidence of association with the other outcomes.

**Conclusions and Relevance:**

In this cohort study of mother-child dyads, adherence to a healthy prenatal dietary pattern was associated with a lower odds of autism diagnosis and social communication difficulties but not restrictive and repetitive behaviors.

## Key Points

**Question:**

What is the association between a healthy prenatal dietary pattern and offspring autism diagnosis and autism-associated traits in 2 large prospective cohort studies: the Norwegian Mother, Father, and Child Cohort (MoBa) and the Avon Study of Parents and Children (ALSPAC)?

**Findings:**

In this cohort study including 84 548 mother-infant dyads in MoBa and 11 670 mother-infant dyads in ALSPAC, maternal consumption of a healthy dietary pattern was associated with reduced likelihood of offspring autism diagnosis (MoBa) and reduced likelihood of social communication difficulties (MoBa and ALSPAC). No other consistent associations were observed.

**Meaning:**

These findings highlight the association between prenatal diet and offspring autism-related outcomes and contribute to the evolving understanding of autism etiology.

## Teaser

This cohort study uses data from 2 large mother-infant cohorts to assess associations of prenatal dietary patterns with offspring autism diagnosis and autism-associated traits up to age 8 years.

## Introduction

The prevalence of autism spectrum disorder diagnosis is estimated to be 1% to 2% in the general population.^1,2^ In this study, we use *autism*, in response to the preferences of the autistic community.^3^ Autism diagnosis reflects a heterogeneous spectrum of neurodevelopmental conditions characterized by persistent difficulties and differences in reciprocal social communication and restricted and repetitive behaviors and interests.^4^ These autism-associated traits extend to subclinical manifestations commonly referred to as the *broader autism phenotype*^5^ and are key areas of development with relevance across the broader population.^6^ Furthermore, the relationship between social communication difficulties and restrictive and repetitive behaviors are phenotypically and genetically dissociable.^7^ Therefore, it may be advantageous to etiological understanding to measure autism diagnosis and autism-associated traits in the population, including the subdomains of social communication difficulties and restrictive and repetitive behaviors.

Prenatal dietary patterns are an emerging plausible exposure in the complex etiology of autism, yet this evidence base is limited. Previous studies have largely focused on discrete facets of prenatal nutrition and found autism diagnosis and autism-associated traits were inversely associated with prenatal multivitamin and/or folic acid supplement use, adequate vitamin D status, and high prenatal fish intakes.^8^ Yet, nutrients have synergistic and antagonistic effects, the summation of which can be measured through prenatal dietary patterns, which could broaden our etiological perspective and complement investigations of discrete nutrients and prenatal multinutrient supplements.^9^

To our knowledge, only 4 studies have investigated the associations of prenatal dietary patterns with autism diagnosis or autism-associated traits. However, the sample sizes were small^10,11,12,13^ and results may have been affected by recall bias.^12,13^ Small sample sizes can increase the risk of type I and type II errors and inflated effect estimates. Furthermore, imprecise measures of diet and autism-associated traits increase random error, which requires larger sample sizes to detect an association, should one exist. Thus, while each study had strengths, their limitations may create heterogeneous results. Therefore, we sought to build on this evidence and measured the associations of high adherence to a healthy prenatal dietary pattern, compared with low adherence, with autism diagnosis and autism-associated traits in 2 large prospective cohort studies, the Norwegian Mother, Father, and Child Cohort Study (MoBa), and the Avon Longitudinal Study of Parents and Children (ALSPAC).

## Methods

### Study Population

The study is reported following the Strengthening the Reporting of Observational Studies in Epidemiology—Nutritional Epidemiology (STROBE-nut) extension of the STROBE statement. We separately analyzed ALSPAC and MoBa to test the consistency of results across contexts, although to ease comparability, we harmonized the analytical approach used in each cohort, where possible. MoBa is a population-based pregnancy cohort study conducted by the Norwegian Institute of Public Health.^14^ Participants were recruited from all over Norway from 1999 to 2008. Of 277 702 eligible pregnancies, 95 200 pregnant individuals (41%) consented to participation. The cohort includes approximately 114 500 children, 95 200 mothers, and 75 200 fathers. Our study is based on version 12 of the quality-assured data files released for research in January 2019. The establishment of MoBa and initial data collection were based on a license from the Norwegian Data Protection Agency and approval from the Regional Committees for Medical and Health Research Ethics. All participants provided written informed consent. The MoBa cohort is currently regulated by the Norwegian Health Registry Act. Our use of MoBa data was approved by the Regional Committees for Medical and Health Research Ethics.

ALSPAC is a prospective cohort primarily of pregnant women and their offspring. Pregnant individuals resident in the Southwest of England with expected dates of delivery between April 1, 1991, and December 31, 1992. Of 20 248 eligible pregnancies, 14 541 pregnant individuals (71.8%) participated in ALSPAC. Further details have been published elsewhere.^15,16^ Details of all the data are available through a fully searchable data dictionary.^17^ Ethical approval for the study was obtained from the ALSPAC Ethics and Law Committee and the local research ethics committees. Informed consent for the use of data collected via questionnaires was implied by return of a completed postal questionnaire following the recommendations of the ALSPAC Ethics and Law Committee at the time.

**Figure 1.**
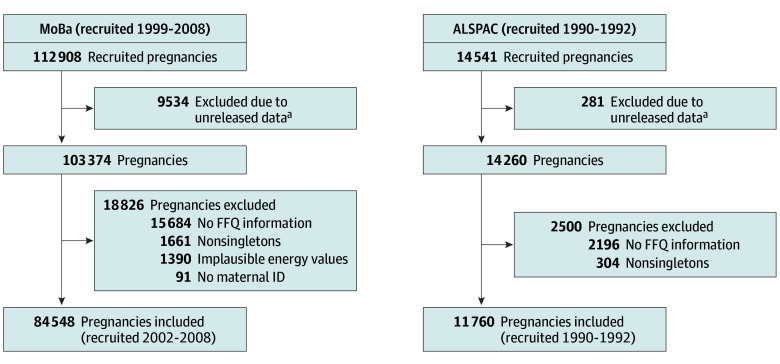
Flowchart of Sample Selection in Norwegian Mother, Father, and Child Cohort (MoBa) and Avon Longitudinal Study of Parents and Children (ALSPAC) FFQ indicates food frequency questionnaire; ID, identifier. ^a^The rationale of unreleased data is detailed in the cohort profiles.^14,15,16^

**Figure 2.**
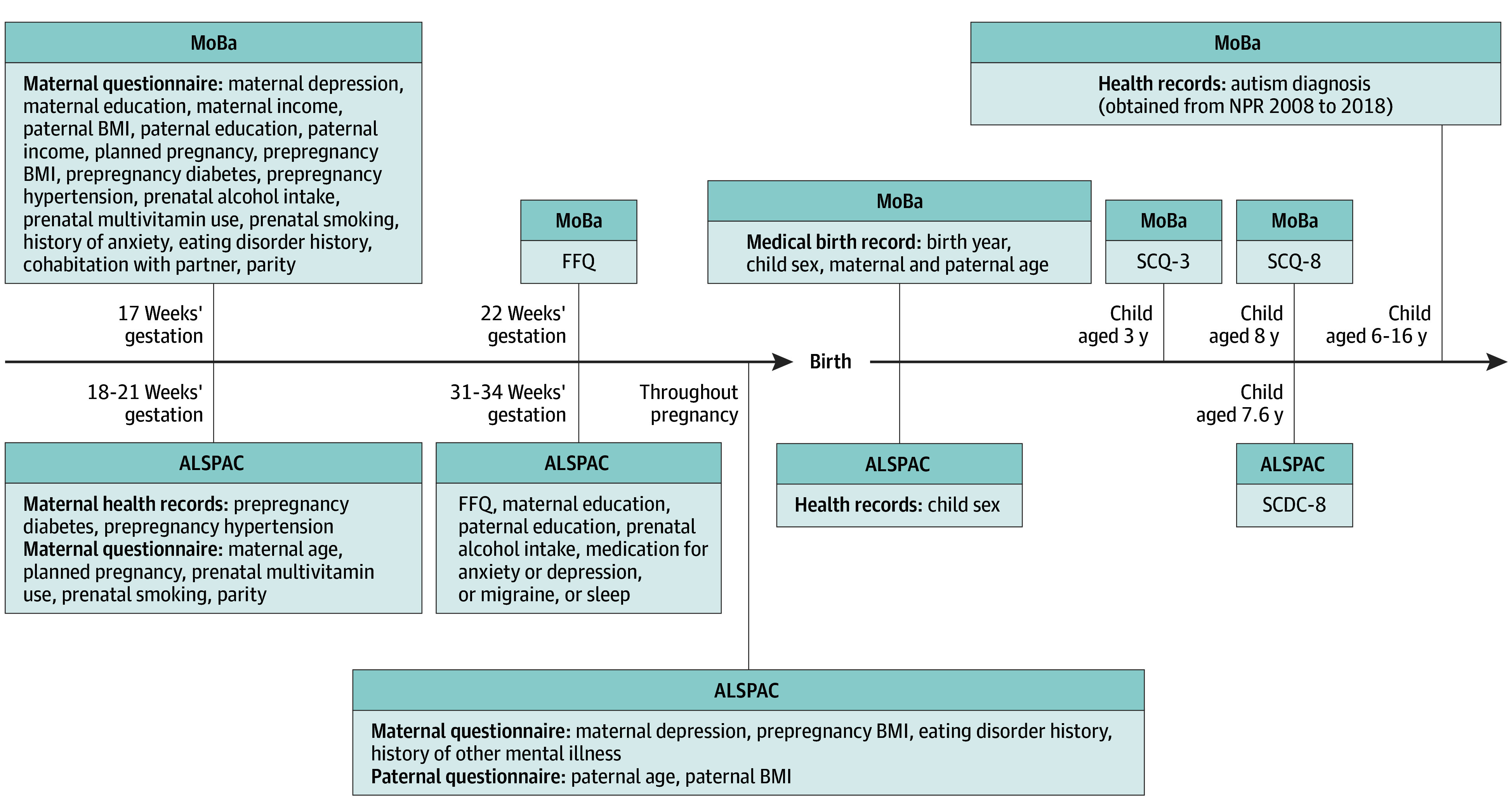
Timeline and Source of Data Collection in Norwegian Mother, Father, and Child Cohort (MoBa) and Avon Longitudinal Study of Parents and Children (ALSPAC) BMI indicates body mass index; FFQ, food frequency questionnaire; NPR, Norwegian patient register; SCDC, Social Communication Disorders Checklist; SCQ, Social Communication Questionnaire.

For our use of MoBa and ALSPAC data, we restricted to plausible food frequency questionnaire (FFQ) responses and singleton pregnancies, which left 84 548 pregnancies in MoBa and 11 760 pregnancies in ALSPAC (Figure 1). In MoBa, FFQ responses were only available for recruitment years 2002 to 2008. Figure 2 shows the source and timeline of all data collection.

### Outcomes

#### Autism Diagnosis

The Norwegian Patient Registry was linked to all MoBa children via unique identification number, capturing all diagnoses of autism made in the public specialist health care system from 2008 to 2018. Children were up to age 16 years at diagnosis. Autism diagnosis is recorded using the *International Statistical Classification of Diseases and Related Health Problems, Tenth Revision* (*ICD-10*) criteria, and we included all F84 codes except Rett syndrome (F84.2).

#### Autism-Associated Traits

From MoBa, we used the 40-item Social Communication Questionnaire (SCQ) about autism-associated social communication difficulties (SCQ-SOC) and restrictive and repetitive behaviors (SCQ-RRB).^18^ The SCQ was completed by the mother about their child at 2 time points, ages 3 and 8 years. Item 1 of the questionnaire screens for phrase speech and is not scored, and we restricted to those with more than 50% response rate.^19^ We applied an adjustment for absence of phrase-speech as per Eaves et al,^20^ as children without phrase-speech may be underscored due to omission of inapplicable items. As secondary outcomes, we analyzed the social communication and restrictive and repetitive behaviors subdomains. From ALSPAC we used the 12-item Social and Communication Disorders Checklist (SCDC), which was completed by the primary caregiver about their child at approximately age 7.6 years. The SCDC is a questionnaire that measures difficulties with social and communication skills. High scores indicated greater autism-associated traits and were defined as at least 93rd percentile for SCDC at age approximately 8 years, SCQ at age 3 years, and SCQ at age 8 years and was guided by previous validation studies for MoBa^19^ and ALSPAC.^21^

### Dietary Assessment and Dietary Pattern Analyses

Both MoBa and ALSPAC used a self-reported FFQ for which detailed methods have been previously published. In MoBa, pregnant participants were asked to report their typical diet since conception in a validated 255-item semiquantitative FFQ.^22,23,24,25^ ALSPAC applied a nonquantitative FFQ that asked pregnant participants to report their current food intake in relation to 43 food groups.^26,27^ MoBa and ALSPAC collected information on commonly consumed foods and beverages and dietary supplement use. Food intake was expressed as frequency of consumption in ALSPAC and grams per day in MoBa.

We matched the food and beverage items used in ALSPAC and MoBa and derived a healthy dietary pattern from each cohort using exploratory factor analysis with varimax rotation (eAppendix 1, eTable 1, and eTable 2 in Supplement 1). The derived factors were selected based on interpretability and Scree plot with eigen values greater than 1.5. The linear factor score was adjusted for total energy intake using the residuals method^28^ and categorized into terciles as high, medium, and low adherence to a healthy prenatal dietary pattern (HPDP). The pattern was denoted healthy as food items with high factor scores represent foods that are encouraged according to dietary guidelines worldwide and nationally.^29,30^ The dietary patterns generally loaded highly for fruits, vegetables, fish, nuts, and whole grain foods and had low factor loadings for red and processed meats, soft drinks, and foods high in fats and/or refined carbohydrates. Additionally, nutrient intakes across the levels of adherence to HPDP (eTable 3 in Supplement 1) were broadly consistent with each country’s respective national nutritional recommendations. See eAppendix 1 in Supplement 1 for further details.

### Statistical Analysis

The analytic strategy was planned a priori and conducted separately in ALSPAC and MoBa; however, the same analytic approach was applied. Descriptive statistics were presented for both the cohorts overall and by tercile of HPDP.

Potential confounders were identified with a directed acyclic graph^31,32^ (eFigure 1 and eAppendix 2 in Supplement 1) and existing literature.^1^ Covariates were maternal age, maternal education, maternal depression, prepregnancy body mass index (BMI; calculated as weight in kilograms divided by height in meters squared), planned pregnancy, prenatal multivitamin use, prenatal alcohol intake, prenatal smoking, prepregnancy hypertension, and prepregnancy diabetes (Table 1; eAppendix 3 in Supplement 1). The minimally sufficient adjustment set was adjusted for using inverse probability weights estimates through entropy balancing.^33^ Weights and covariate balance were checked (<0.1 negligible covariate imbalance).^34^ Within a generalized nonlinear model, we further clustered on the mother to account for the intercorrelation between siblings. Robust standard errors were estimated using Horvitz-Thompson variance estimator to account for the inverse probability weights and clustering.^35,36^ All statistical models were 2-tailed, with α = .05. We used R Studio version 4.0.3 (for MoBa analyses and version 4.2.2 for ALSPAC data (R Project for Statistical Computing) to conduct analyses, along with several key packages.^37,38,39^ Data analysis occurred February 1, 2022, to August 1, 2023.

**Table 1.** Sociodemographic Characteristics by Adherence to a Healthy Prenatal Dietary Pattern in MoBa and ALSPAC

Variable	Participants, No. (%)							
	MoBa				ALSPAC			
	Overall (N = 84 548)	HPDP adherence			Overall (N = 11 760)	HPDP adherence		
		Low (n = 28 183)	Medium (n = 28 182)	High (n = 28 183)		Low (n = 3920)	Medium (n = 3920)	High (n = 3920)
Child sex								
Male	43 277 (51.2)	14 465 (51.3)	14 443 (51.2)	14 369 (51.0)	6034 (51.3)	2017 (51.5)	1998 (51.0)	2019 (51.5)
Female	41 206 (48.7)	13 690 (48.6)	13 717 (48.7)	13 799 (49.0)	5725 (48.7)	1902 (48.5)	1922 (49.0)	1901 (48.5)
Missing	65 (0.1)	28 (0.1)	22 (0.1)	15 (0.1)	1 (<0.1)	1 (<0.1)	0	0
Maternal history of depression								
No	76 340 (90.3)	25 331 (89.9)	25 604 (90.9)	25 405 (90.1)	10 263 (87.3)	3261 (83.2)	3470 (88.5)	3532 (90.1)
Yes	6474 (7.7)	2262 (8.0)	2021 (7.2)	2191 (7.8)	956 (8.1)	391 (10.0)	282 (7.2)	283 (7.2)
Missing	1734 (2.1)	590 (2.1)	557 (2.0)	587 (2.1)	541 (4.6)	268 (6.8)	168 (4.3)	105 (2.7)
Maternal age								
Mean (SD), y	30.2 (4.6)	29.1 (4.6)	30.4 (4.4)	31.3 (4.5)	27.9 (4.9)	26.0 (4.7)	27.9 (4.64)	29.8 (4.4)
Missing	0	0	0	0	250 (2.1)	131 (3.3)	75 (1.9)	44 (1.1)
Maternal education level^a^								
1	1960 (2.3)	974 (3.5)	546 (1.9)	440 (1.6)	2324 (19.8)	1311 (33.4)	698 (17.8)	315 (8.0)
2	3736 (4.4)	1851 (6.6)	1057 (3.8)	828 (2.9)	1155 (9.8)	522 (13.3)	405 (10.3)	228 (5.8)
3	10 253 (12.1)	4651 (16.5)	3271 (11.6)	2331 (8.3)	4063 (34.5)	1432 (36.5)	1533 (39.1)	1098 (28.0)
4	11 267 (13.3)	4651 (16.5)	3538 (12.6)	3078 (10.9)	2645 (22.5)	498 (12.7)	887 (22.6)	1260 (32.1)
5	33 190 (39.3)	10 617 (37.7)	11 698 (41.5)	10 875 (38.6)	1503 (12.8)	119 (3.0)	377 (9.6)	1007 (25.7)
6	19 319 (22.8)	3822 (13.6)	6550 (23.2)	8947 (31.7)	NA	NA	NA	NA
Missing	4823 (5.7)	1617 (5.7)	1522 (5.4)	1684 (6.0)	70 (0.6)	38 (1.0)	20 (0.5)	12 (0.3)
Planned pregnancy								
No	15 512 (18.3)	5642 (20.0)	4819 (17.1)	5051 (17.9)	3356 (28.5)	1271 (32.4)	1099 (28.0)	986 (25.2)
Yes	67 461 (79.8)	22 036 (78.2)	22 884 (81.2)	22 541 (80.0)	8113 (69.0)	2504 (63.9)	2731 (69.7)	2878 (73.4)
Missing	1575 (1.9)	505 (1.8)	479 (1.7)	591 (2.1)	291 (2.5)	145 (3.7)	90 (2.3)	56 (1.4)
Prenatal alcohol intake								
No	64 040 (75.7)	21 874 (77.6)	21 386 (75.9)	20 780 (73.7)	4524 (38.5)	1627 (41.5)	1539 (39.3)	1358 (34.6)
Yes	8861 (10.5)	2577 (9.1)	3045 (10.8)	3239 (11.5)	2130 (18.1)	538 (13.7)	704 (18.0)	888 (22.7)
Missing	11 647 (13.8)	3732 (13.2)	3751 (13.3)	4164 (14.8)	5106 (43.4)	1755 (44.8)	1677 (42.8)	1674 (42.7)
Prenatal multivitamin supplement use								
No	55 706 (65.9)	20 282 (72.0)	18 268 (64.8)	17 156 (60.9)	8877 (75.5)	3067 (78.2)	3009 (76.8)	2801 (71.5)
Yes	24 632 (29.1)	6506 (23.1)	8551 (30.3)	9575 (34.0)	2568 (21.8)	683 (17.4)	823 (21.0)	1062 (27.1)
Missing	4210 (5.0)	1395 (4.9)	1363 (4.8)	1452 (5.2)	315 (2.7)	170 (4.3)	88 (2.2)	57 (1.5)
Prenatal smoking								
No	70 442 (83.3)	21 843 (77.5)	23 815 (84.5)	24 784 (87.9)	8821 (75.0)	2426 (61.9)	3033 (77.4)	3362 (85.8)
Yes	6275 (7.4)	3333 (11.8)	1752 (6.2)	1190 (4.2)	2709 (23.0)	1368 (34.9)	820 (20.9)	521 (13.3)
Missing	7831 (9.3)	3007 (10.7)	2615 (9.3)	2209 (7.8)	230 (2.0)	126 (3.2)	67 (1.7)	37 (0.9)
Prepregnancy BMI^b^								
Mean (SD)	24.1 (4.3)	24.5 (4.6)	24.1 (4.2)	23.5 (4.0)	22.9 (3.8)	23.3 (4.3)	23.1 (3.79)	22.4 (3.3)
<18.5	2397 (2.8)	872 (3.1)	685 (2.4)	840 (3.0)	500 (4.3)	198 (5.1)	148 (3.8)	154 (3.9)
18.5-24.9	53 635 (63.4)	16 558 (58.8)	17 826 (63.3)	19 251 (68.3)	7775 (66.1)	2303 (58.8)	2545 (64.9)	2927 (74.7)
25-29.9	17 865 (21.1)	6537 (23.2)	6165 (21.9)	5163 (18.3)	1578 (13.4)	588 (15.0)	580 (14.8)	410 (10.5)
≥30	7843 (9.3)	3281 (11.6)	2604 (9.2)	1958 (6.9)	564 (4.8)	247 (6.3)	197 (5.0)	120 (3.1)
Missing	2808 (3.3)	935 (3.3)	902 (3.2)	971 (3.4)	1343 (11.4)	584 (14.9)	450 (11.5)	309 (7.9)
Prepregnancy diabetes								
No	82 401 (97.5)	27 501 (97.6)	27 484 (97.5)	27 416 (97.3)	10 706 (91.0)	3454 (88.1)	3592 (91.6)	3660 (93.4)
Yes	413 (0.5)	92 (0.3)	141 (0.5)	180 (0.6)	44 (0.4)	13 (0.3)	15 (0.4)	16 (0.4)
Missing	1734 (2.1)	590 (2.1)	557 (2.0)	587 (2.1)	1010 (8.6)	453 (11.6)	313 (8.0)	244 (6.2)
Prepregnancy hypertension								
No	82 216 (97.2)	27 411 (97.3)	27 455 (97.4)	27 350 (97.0)	11 663 (99.2)	3883 (99.1)	3889 (99.2)	3891 (99.3)
Yes	882 (1.0)	309 (1.1)	281 (1.0)	292 (1.0)	28 (0.2)	9 (0.2)	7 (0.2)	12 (0.3)
Missing	1450 (1.7)	463 (1.6)	446 (1.6)	541 (1.9)	69 (0.6)	28 (0.7)	24 (0.6)	17 (0.4)

Abbreviations: ALSPAC, Avon Longitudinal Study of Parents and Children; BMI, body mass index (calculated as weight in kilograms divided by height in meters squared); HPDP, healthy prenatal dietary pattern; MoBa, Norwegian Mother, Father, and Child Cohort; NA, not applicable.

^a^ Level 1 was defined as less than 9 years of elementary education (MoBa) or certificate of secondary education or none (ALSPAC); 2, 1 to 2 years of further education (MoBa) and vocational school (ALSPAC); 3, technical high school (MoBa) or ordinary level (ALSPAC); 4, 3-year high school general studies or junior college (MoBa) or advanced level (ALSPAC); 5, regional technical college or 4-year university degree (Bachelor’s degree, nurse, teacher, engineer) (MoBa) or a college degree (ALSPAC); and 6, more than 4 years at university or technical college (Master’s degree, physician, PhD) (MoBa).

^b^ Prepregnancy BMI was modeled linearly but BMI categories are presented descriptively to aid interpretation.

Data were assumed to be missing at random and imputed using multivariate imputation by chained equations.^38^ We included all variables in the models and a range of auxiliary variables (eTable 4 in Supplement 1). We used 115 imputations for ALSPAC and 156 imputations for MoBa, selected based on the von Hippel approach,^40^ and imputed each level of HPDP separately to facilitate the testing of interaction terms.^41^ The weights and marginal structural models were estimated for each imputed dataset^42^ before combining them using Rubin rule to produce the single and final estimate of association.^43^ The characteristics of participants with missing outcome information are presented in eTables 5 to 7 in Supplement 1.

In sensitivity analyses, we repeated the main analyses making changes to the confounding, outcome, and exposure measurements (eAppendix 4 in Supplement 1). For confounding, we adjusted for additional covariates, including paternal characteristics and birth year, and assessed the estimates without adjustment for prenatal multivitamin supplement use. For sensitivity analysis of outcomes, we applied an alternative approach to score the SCQ and described the overlap in children with high scores for SCQ at ages 3 and 8 years and autism diagnosis. For sensitivity analysis of our exposure, we replaced the HPDP with each of its subgroups, plant-based, fish-based, and unhealthy dietary patterns (eTable 2 in Supplement 1). Interactions tested were between HPDP and prepregnancy BMI (<25 vs ≥25), child’s sex (male or female), multivitamin supplement use (yes or no) and maternal education (MoBa: less than college or university degree or a degree or higher; ALSPAC: less than A-level or greater than A-level). Lastly, complete-case analyses were conducted.

## Results

MoBa included 84 548 pregnancies (mean [SD] age, 30.2 [4.6] years; 43 277 [51.2%] male offspring) and ALSPAC had 11 760 pregnancies (mean [SD] age, 27.9 [4.7] years; 6034 [51.3%] male offspring). The sociodemographic characteristics varied across cohorts and within cohorts (Table 1; eTable 8 in Supplement 1). Compared with ALSPAC participants, MoBa participants were older at recruitment, had higher educational levels, and were more likely to have planned their pregnancy, abstain from alcohol, and use a multivitamin supplement. MoBa and ALSPAC participants with high HPDP adherence, compared with those with low adherence, were more likely to be older, with high educational attainment, use prenatal multivitamin supplements, and be nonsmokers. In ALSPAC only, high HPDP adherence was associated with greater prevalence of alcohol consumption in pregnancy and lower prevalence of a planned pregnancy and history of depression. Good covariate balance was achieved, and there were no extreme weights (eFigure 2 and eFigure 3 in Supplement 1).

The event rate for each outcome (autism diagnosis from SCQ at ages 3 and 8 years or SCDC at age 8 years) was only higher in the lowest level of adherence to a HPDP compared with medium or high adherence (Table 2). The proportions of children with recurrent high scores across the outcomes were modest but was especially low for SCQ-RRB at age 3 years (eTable 9 in Supplement 1).

**Table 2.** Autism Diagnosis and Autism-Related Traits by Adherence to a Healthy Prenatal Dietary Pattern in ALSPAC and MoBa

Outcome	Participants by maternal adherence to a healthy prenatal dietary pattern, No. (%)			
	Overall	Low	Medium	High
**MoBa**				
Pregnancies, No.	84 548	28 183	28 182	28 183
Autism diagnosis				
No	83 606 (98.9)	27 791 (98.6)	27 901 (99.0)	27 914 (99.0)
Yes	942 (1.1)	392 (1.4)	281 (1.0)	269 (1.0)
Missing	0	0	0	0
Age 3 y				
SCQ^a^				
No	46 154 (54.6)	15 017 (53.3)	15 645 (55.5)	15 492 (55.0)
Yes	5408 (6.4)	1879 (6.7)	1736 (6.2)	1793 (6.4)
Missing	32 986 (39.0)	11 287 (40.0)	10 801 (38.3)	10 898 (38.7)
SCQ-RRB^a^				
No	47 078 (55.7)	15 456 (54.8)	15 978 (56.7)	15 644 (55.5)
Yes	4484 (5.3)	1440 (5.1)	1403 (5.0)	1641 (5.8)
Missing	32 986 (39.0)	11 287 (40.0)	10 801 (38.3)	10 898 (38.7)
SCQ-SOC^a^				
No	46 481 (55.0)	14 961 (53.1)	15 678 (55.6)	15 842 (56.2)
Yes	5081 (6.0)	1935 (6.9)	1703 (6.0)	1443 (5.1)
Missing	32 986 (39.0)	11 287 (40.0)	10 801 (38.3)	10 898 (38.7)
Age 8 y				
SCQ^a^				
No	36 786 (43.5)	11 677 (41.4)	12 460 (44.2)	12 649 (44.9)
Yes	3468 (4.1)	1196 (4.2)	1184 (4.2)	1088 (3.9)
Missing	44 294 (52.4)	15 310 (54.3)	14 538 (51.6)	14 446 (51.3)
SCQ-RRB^a^				
No	34 237 (40.5)	10 864 (38.5)	11 662 (41.4)	11 711 (41.6)
Yes	6017 (7.1)	2009 (7.1)	1982 (7.0)	2026 (7.2)
Missing	44 294 (52.4)	15 310 (54.3)	14 538 (51.6)	14 446 (51.3)
SCQ-SOC^a^				
No	37 218 (44.0)	11 829 (42.0)	12 598 (44.7)	12 791 (45.4)
Yes	3036 (3.6)	1044 (3.7)	1046 (3.7)	946 (3.4)
Missing	44 294 (52.4)	15 310 (54.3)	14 538 (51.6)	14 446 (51.3)
**ALSPAC**				
Participants, No.	11 760	3920	3920	3920
SCDC at age 8 y^a^				
No	6735 (57.3)	1865 (47.6)	2274 (58.0)	2596 (66.2)
Yes	544 (4.6)	210 (5.4)	160 (4.1)	174 (4.4)
Missing	4481 (38.1)	1845 (47.1)	1486 (37.9)	1150 (29.3)

Abbreviations: ALSPAC, Avon Longitudinal Study of Parents and Children; MoBa, Norwegian Mother, Father, and Child Cohort; SCDC, Social Communication Disorders Checklist; SCQ, Social Communication Questionnaire; SCQ-RRB, restrictive and repetitive behaviors domain of SCQ; SCQ-SOC, communication skills domain of SCQ.

^a^ The presence of the outcome from each questionnaire was indicated by a high score.

**Figure 3.**
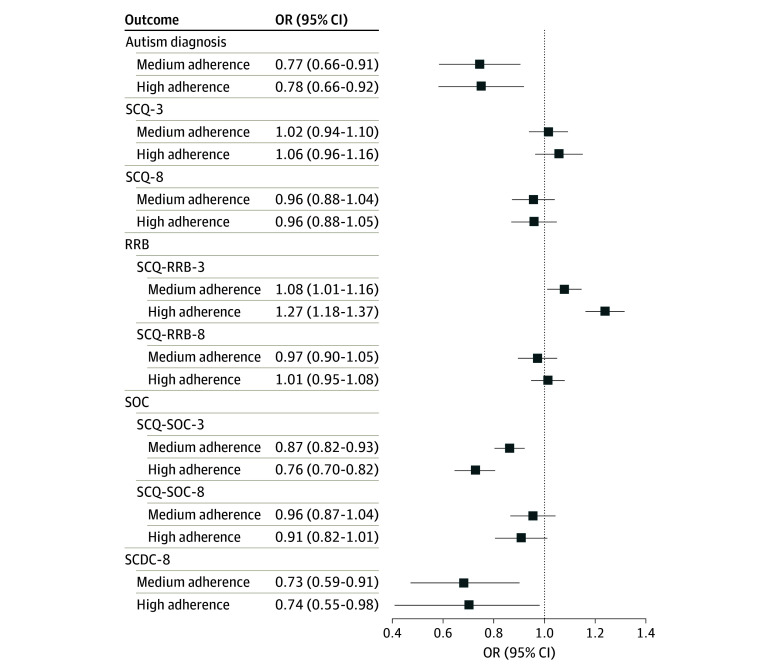
Associations Between Adherence to a Healthy Prenatal Dietary Pattern and Autism-Related Outcomes Adjusted covariates in each model were child sex, maternal history of depression, maternal age, maternal education, planned pregnancy, prenatal alcohol intake, prenatal multivitamin supplement use, prenatal smoking, prepregnancy body mass index, prepregnancy diabetes, and prepregnancy hypertension. The reference category was low adherence to a healthy prenatal dietary pattern. Autism was measured as a binary outcome (yes or no), and the other outcomes represent high levels of autism-related traits, including restrictive and repetitive behaviors (RRB) and social communication difficulties (SOC). SCDC indicates Social Communication Disorders Checklist; SCQ, Social Communication Questionnaire. The number following each outcome denotes the approximate age of the child in years when the measure was obtained eg, SCQ-8 Social Communication Questionnaire measured at age 8 years. The presence of the outcome from each questionnaire was indicated by a high score. OR indicates odds ratio.

There were lower odds of each outcome in the crude models in association with HPDP (autism diagnosis: OR, 0.68 [95% CI, 0.61-0.83]; SCQ at age 3 years: OR, 0.86 [95% CI, 0.79-0.93]; SCQ at age 8 years: OR, 0.80 [95% CI, 0.72-0.88]; SCDC at age 8 years: OR, 0.59 [95% CI, 0.48-0.74]) (eTable 10 in Supplement 1). Higher HPDP adherence was associated with a lower likelihood of autism diagnosis in MoBa and lower SCDC score at age 8 years in ALSPAC, yet no clear evidence of association was observed for SCQ at ages 3 or 8 years in MoBa (Figure 3). In MoBa, higher HPDP adherence was associated with a lower likelihood of a high score on SCQ-SOC at age 3 years but higher likelihood of a high score on SCQ-RRB at age 3 years. Results were robust to adjustment for additional covariates (eTable 11 in Supplement 1).

Further sensitivity analysis indicated that, generally, the clearest associations in HPDP were in analyses of the food subgroups. The likelihood of a high score on SCQ at age 8 years (OR, 0.92 [95% CI, 0.86-0.99]), and SCQ-SOC at age 8 years (OR, 0.89 [95% CI, 0.82-0.96]) reduced in association with a plant-based dietary pattern, and the likelihood of a high score on SCQ-RRB at age 8 years (OR, 0.93 [95% CI, 0.88-0.99]) reduced in association with a fish-based dietary pattern. Compared with the HPDP analysis, the differences were small (eTable 12 in Supplement 1). Evidence for interaction was unclear and/or inconsistent across MoBa and ALSPAC, except between HPDP and child sex (SCQ-SOC at age 3 years, SCQ-SOC at age 8 years, and SCDC at age 8 years) (eTable 13 in Supplement 1). A greater magnitude of association was observed in female offspring in relation to SCQ-SOC at age 8 years and SCDC at age 8 years and for male offspring in relation to SCQ-SOC at age 3 years. The complete-case analyses in MoBa produced similar results to the main analyses (eTable 14 in Supplement 1).

## Discussion

Our cohort study found a lower likelihood of autism diagnosis and autism-associated traits for offspring of mothers with high adherence to HPDP, albeit inconsistently for measures of autism-associated traits. This inconsistency related to differential associations with the subdomains and at different ages. Overall, more consistent results were observed for social communication difficulties. Lastly, there was weak evidence of association modification by child sex, but inconsistent evidence with the other characteristics, maternal education, prepregnancy BMI, and prenatal supplement use.

Our large prospective investigation makes an important contribution to existing literature. Previous studies have largely focused on multinutrient supplements, discrete nutrients, or food groups.^8^ Yet, diet makes the largest contribution to overall nutrient intakes and captures the totality of complex nutrient interactions.^9^ Moreover, there is a small body of existing literature on prenatal diet and autism-associated outcomes, and results are conflicting. We addressed some limitations of previous investigations, such as small sample size and retrospective study design, so that our analyses may provide more reliable results.

Two retrospective studies found a healthy prenatal diet was associated with a lower likelihood of autism diagnosis,^12,13^ but a prospective investigation did not replicate their findings.^10^ However, the retrospective investigations had a high risk of bias, especially recall bias,^12,13^ and most studies included fewer than 100 children with autism diagnosis.^10,12^ We only identified 2 previous prospective investigations into autism-associated traits (measured linearly), but their results were conflicting. The first study investigated 2 cohorts with 727 and 154 mother-child dyads and measured offspring social responsiveness scores between ages 4 to 18 years and 36 months, respectively.^10^ Of the 6 prenatal dietary patterns investigated, none were clearly associated with autism-associated traits in the final adjusted model.^10^ The second prospective study, including 325 mother-child dyads, observed less autism-associated traits at age 12 to 14 months if mothers had a greater Mediterranean dietary pattern score. Autism-associated traits were measured using an adapted version of the Infant Toddler Social and Emotional Assessment questions.^11^ We obtained more precise estimates, yet our main results on autism-associated traits were also conflicting.

We considered whether the subdomains or child’s age at measurement were potential sources of heterogeneity. The variable findings on autism-associated traits may relate to the performance of the SCQ at ages 3 or 8 years and SCDC at age 8 years. In addition to autism diagnosis, the SCQ and SCDC detect children with greater social communication difficulties or inflexible behaviors and interests that do not necessarily constitute a diagnosable condition.^19,21^ This is particularly true in children younger than 4 years, especially for restrictive and repetitive behaviors, which are a common feature of typical development.^44^ We estimated that only approximately one-fifth of children with a high SCQ score at age 3 years continued to score highly at age 8 years, although this estimate may be underestimated due to missing data at age 8 years. A validation study in MoBa confirmed the SCQ-RRB did not discriminate between autistic and nonautistic children at age 3 years, whereas the SCQ-SOC performed fairly well.^19^ Additionally, we compared the full SCQ (39 items) and SCDC, as they are designed to screen for autism,^19,21^ yet the SCDC only measures social communication skills.^21^ Overall, social communication difficulties were more consistently associated with HPDP.

We observed the association of HPDP with social communication skills in both cohorts, despite variability in the composition of HPDP between MoBa and ALSPAC. Furthermore, the findings on autism were consistent with 3 other studies that applied various approaches to measure dietary patterns and autism outcomes.^11,12,13^ At a population level, dietary patterns provide a broad approximation of a healthy diet that averages across the rich aspects of diet, measured and unmeasured.^45,46,47^ Similar consistency in diet-outcome associations across dietary pattern measures are observed in other fields.^45,48^ It has been suggested that this may relate to broadly similar core components of a healthy diet, such as high intakes of fruits, vegetables, and whole grains and low intakes of animal products and highly processed foods.^49^

We observed inconsistent evidence that female offspring, compared with male offspring, had a greater reduction in the likelihood of social communication difficulties at age approximately 8 years if their mothers had consumed an HPDP. This was replicated across MoBa and ALSPAC but only in relation to the highest adherence to HPDP and not at age 3 years. Despite considerable interest in a theorized female protective effect, few investigations test for association modification by child sex.^50^ Studies that did observed a larger magnitude of association in females compared with males^51,52,53^ or no clear differences, although their main results were also null.^10,54,55,56^

The male preponderance of autism diagnosis contributes to debates of a female protective effect. Several mechanisms have been proposed and relate to an extreme male brain, sex hormones, genetics, and immune function.^50^ However, misclassification bias and sex differences in developmental trajectories may impact results. The conceptualization of autism has been predominantly based on White males, which has led to underdiagnosis in females and can impact the performance of autism-screening tools.^57^ Additionally, many features of communication develop earlier in girls compared with boys,^57^ which may further explain our conflicting results between ages 3 and 8 years. Overall, social communication skills may be more strongly associated with HPDP in females, but the mechanisms remain to be determined.

Lastly, although nutrients are distributed across multiple foods, there are key food sources of specific nutrients. In MoBa, and to a lesser extent in ALSPAC, plant-based foods, compared with fish-based and unhealthy foods, had clearer associations with social communication difficulties. There are several explanations for the variation across food groups, such as differences in random error and bias structures, larger number of food items, or a true effect of plant-based foods. We identified a previous study that directly compared food groups while simultaneously adjusting for other foods; however, the results were inconclusive, possibly due to limited statistical power to detect an association.^58^ Further studies are required to clarify if plant-based foods have clearer associations with social communication difficulties.

Whether the associations observed are causal remains to be established. The etiology of autism has been linked to several pathways, such as genetics, maternal immune activation, sex hormones, the microbiome, and environmental factors.^51^ It is hypothesized that prenatal diet may alter DNA methylation patterns,^11^ regulate immune processes, or interact with toxins.^8^

### Strengths and Limitations

Our study had many strengths, such as the large prospective design, cross context comparison, analyses of subdomains of autism-associated traits, dietary subgroups, and interactions. We carefully considered potential confounders and adjusted for a wide range, including paternal characteristics, and further tested this through sensitivity analysis. However, unmeasured, and residual confounding can still occur, for example, genetic confounding, parental caregiving or childhood diet. A further strength of our investigation was the analysis of detailed dietary information collected prospectively during pregnancy. Nonetheless, autism-associated traits and prenatal diet are complex measures that rely on self-reported information, and FFQ can only provide an imprecise approximation of habitual dietary intake.^22,23,24,25^ We adjusted for a proportion of error through the residuals method.^28^ Additionally, a single assessment of maternal diet is a limitation and precludes the evaluation of dietary changes over time. Although some dietary changes during pregnancy may occur, misclassification would likely bias results toward the null.

Even autism diagnosis is broadly defined, and diagnostic criteria and practices have changed over time.^58^ These factors increase heterogeneity, reduce precision, and increase bias, which may have affected our results. Selection bias may affect our findings through the inevitable exclusion of nonviable pregnancies, systematic differences in recruitment and retention, especially in the autism-associated traits analysis due to the high attrition rate.^14,15^ Mothers with low adherence to HPDP and offspring with high SCQ scores at age 3 years had a greater proportion of missing data for SCQ at age 8 years. This further indicates selection bias, which would bias toward the null and reduce generalizability.

## Conclusions

In this cohort study of mother-child dyads, the likelihood of autism diagnosis was reduced by 22% in association with high adherence to HPDP. Regarding autism-associated traits, we observed an association between social communication difficulties and higher HPDP adherence but inconsistent associations with restrictive and repetitive behaviors. Furthermore, we observed that female offspring may have a greater magnitude of association of adherence to HPDP with social communication difficulties at age 8 years. At present, we remain uncertain as to whether the associations observed are causal. Further research should substantiate our findings, especially given the inconsistency in the previous literature and across our measures of autism-associated traits. It would be advantageous to measure the subdomains combined and separately and explore whether associations differ by food group. Additionally, triangulation with alternative study designs and exploration of potential mediators is required to support causal interpretation of the associations observed in our study.

## Notes

Supplement 1. **eAppendix 1.** Dietary Patterns **eTable 1.** Food Items and Factor Loadings Representing a Healthy Prenatal Dietary Pattern **eTable 2.** Food Items and Factor Loadings of the Subgroups of a Healthy Prenatal Dietary Pattern **eTable 3.** Estimated Nutrient Intakes in ALSPAC and MoBa by Level of Adherence to a Healthy Prenatal Dietary Pattern **eAppendix 2.** Explanation of Directed Acyclic Graph **eFigure 1.** Directed Acyclic Graph of Relationship Between Healthy Prenatal Dietary Pattern and Autism-Related Outcomes **eAppendix 3.** Covariates **eAppendix 4.** Sensitivity Analyses **eTable 4.** Variables Used in Imputation Models **eTable 5.** Baseline Characteristics of Pregnancies With Missing Outcome Data at Age Three in MoBa **eTable 6.** Baseline Characteristics of Pregnancies With Missing Outcome Data at Age Eight in MoBa **eTable 7.** Baseline Characteristics of Pregnancies With Missing Outcome Data at Age Eight in ALSPAC **eTable 8.** Descriptive Statistics for Additional Sociodemographic Characteristics Used in Sensitivity Analyses **eTable 9.** Number and Proportion of Children Across Each Outcome in MoBa **eFigure 2.** Covariate Balance Plot for Adherence to a Healthy Prenatal Dietary Pattern in ALSPAC **eFigure 3.** Covariate Balance Plot for Adherence to a Healthy Prenatal Dietary Pattern in MoBa **eTable 10.** Unadjusted Measures of Association Between a Healthy Prenatal Dietary Pattern and Autism Diagnosis and Autism-Associated Traits **eTable 11.** Associations Between a Healthy Prenatal Dietary Pattern and Autism Diagnosis and Autism-Associated Traits in MoBa and ALSPAC With a Different Adjustment Set of Covariates **eTable 12.** Associations Between a Healthy Prenatal Dietary Pattern and Food Groups With Autism Diagnosis and Autism-Associated Traits in ALSPAC and MoBa **eTable 13.** Associations Between a Healthy Prenatal Dietary Pattern With Autism Diagnosis and Autism-Associated Traits in MoBa and ALSPAC Stratified by Prenatal Multivitamin Supplement Use, Prepregnancy BMI, Child Sex, and Maternal Education **eTable 14.** Complete Case Analyses Measuring the Associations Between Adherence to a Healthy Prenatal Dietary Pattern and Autism Diagnosis and Autism-Associated Traits in MoBa
eReferences.

Supplement 2.
Data Sharing Statement
