# Supplementary material for: Healthy Prenatal Dietary Pattern and Offspring Autism
Source: JAMA Netw Open. 2024 Jul 18;7(7):e2422815. doi: 10.1001/jamanetworkopen.2024.22815 (PMC11258593; doi:10.1001/jamanetworkopen.2024.22815)
Supplement: Supplement 1. — eAppendix 1. Dietary Patterns eTable 1. Food Items and Factor Loadings Representing a Healthy Prenatal Dietary Pattern eTable 2. Food Items and Factor Loadings of the Subgroups of a Healthy Prenatal Dietary Pattern eTable 3. Estimated Nutrient Intakes in ALSPAC and MoBa by Level of Adherence to a Healthy Prenatal Dietary Pattern eAppendix 2. Explanation of Directed Acyclic Graph eFigure 1. Directed Acyclic Graph of Relationship Between Healthy Prenatal Dietary Pattern and Autism-Related Outcomes eAppendix 3. Covariates eAppendix 4. Sensitivity Analyses eTable 4. Variables Used in Imputation Models eTable 5. Baseline Characteristics of Pregnancies With Missing Outcome Data at Age Three in MoBa eTable 6. Baseline Characteristics of Pregnancies With Missing Outcome Data at Age Eight in MoBa eTable 7. Baseline Characteristics of Pregnancies With Missing Outcome Data at Age Eight in ALSPAC eTable 8. Descriptive Statistics for Additional Sociodemographic Characteristics Used in Sensitivity Analyses eTable 9. Number and Proportion of Children Across Each Outcome in MoBa eFigure 2. Covariate Balance Plot for Adherence to a Healthy Prenatal Dietary Pattern in ALSPAC eFigure 3. Covariate Balance Plot for Adherence to a Healthy Prenatal Dietary Pattern in MoBa eTable 10. Unadjusted Measures of Association Between a Healthy Prenatal Dietary Pattern and Autism Diagnosis and Autism-Associated Traits eTable 11. Associations Between a Healthy Prenatal Dietary Pattern and Autism Diagnosis and Autism-Associated Traits in MoBa and ALSPAC With a Different Adjustment Set of Covariates eTable 12. Associations Between a Healthy Prenatal Dietary Pattern and Food Groups With Autism Diagnosis and Autism-Associated Traits in ALSPAC and MoBa eTable 13. Associations Between a Healthy Prenatal Dietary Pattern With Autism Diagnosis and Autism-Associated Traits in MoBa and ALSPAC Stratified by Prenatal Multivitamin Supplement Use, Prepregnancy BMI, Child Sex, and Maternal Education eTable 14. Comp [file jamanetwopen-e2422815-s001.pdf]

Supplemental Online Content

Friel C, Leyland AH, Anderson JJ, Havdahl A, Brantsæter AL, Dundasa R. Healthy prenatal dietary pattern and offspring autism. *JAMA Netw Open*. 2024;7(7):e2422815. doi:10.1001/jamanetworkopen.2024.22815

eAppendix 1. Dietary Patterns
eTable 1. Food Items and Factor Loadings Representing a Healthy Prenatal Dietary Pattern
eTable 2. Food Items and Factor Loadings of the Subgroups of a Healthy Prenatal Dietary Pattern
eTable 3. Estimated Nutrient Intakes in ALSPAC and MoBa by Level of Adherence to a Healthy Prenatal Dietary Pattern
eAppendix 2. Explanation of Directed Acyclic Graph
eFigure 1. Directed Acyclic Graph of Relationship Between Healthy Prenatal Dietary Pattern and Autism-Related Outcomes
eAppendix 3. Covariates
eAppendix 4. Sensitivity Analyses
eTable 4. Variables Used in Imputation Models
eTable 5. Baseline Characteristics of Pregnancies With Missing Outcome Data at Age Three in MoBa
eTable 6. Baseline Characteristics of Pregnancies With Missing Outcome Data at Age Eight in MoBa
eTable 7. Baseline Characteristics of Pregnancies With Missing Outcome Data at Age Eight in ALSPAC
eTable 8. Descriptive Statistics for Additional Sociodemographic Characteristics Used in Sensitivity Analyses
eTable 9. Number and Proportion of Children Across Each Outcome in MoBa
eFigure 2. Covariate Balance Plot for Adherence to a Healthy Prenatal Dietary Pattern in ALSPAC
eFigure 3. Covariate Balance Plot for Adherence to a Healthy Prenatal Dietary Pattern in MoBa
eTable 10. Unadjusted Measures of Association Between a Healthy Prenatal Dietary Pattern and Autism Diagnosis and Autism-Associated Traits
eTable 11. Associations Between a Healthy Prenatal Dietary Pattern and Autism Diagnosis and Autism-Associated Traits in MoBa and ALSPAC With a Different Adjustment Set of Covariates
eTable 12. Associations Between a Healthy Prenatal Dietary Pattern and Food Groups With Autism Diagnosis and Autism-Associated Traits in ALSPAC and MoBa
eTable 13. Associations Between a Healthy Prenatal Dietary Pattern With Autism Diagnosis and Autism-Associated Traits in MoBa and ALSPAC Stratified by Prenatal Multivitamin Supplement Use, Prepregnancy BMI, Child Sex and Maternal Education
eTable 14. Complete Case Analyses Measuring the Associations Between Adherence to a Healthy Prenatal Dietary Pattern and Autism Diagnosis and Autism-Associated Traits in MoBa
eReferences.

This supplemental material has been provided by the authors to give readers additional information about their work.

© 2024 Friel C et al. *JAMA Network Open.*

eAppendix 1. Dietary patterns

Factor loadings are the correlation coefficients between the factor (HPDP) and food item which reflect the relative importance of foods within a HPDP, rather than quantity. The value of the factor loading is therefore sensitive to the number of items within the model and are not expected to be the exact same between MoBa and ALSPAC. Nonetheless, there are likely to be differences in the underlying diets between each cohort. In MoBa and ALSPAC, a HPDP loaded highly for fruits, vegetables, nuts, lean and oily fish, and low in fries and processed meats (eTable 1). However, high fibre bread loaded positively in MoBa but negatively in ALSPAC, and soft drinks loaded negatively in MoBa but were unimportant in ALSPAC.

We compared the factor loadings observed in MoBa and ALSPAC in the context of cultural trends in diet, around the time of pregnancy. The results were consistent with dietary trends observed in the respective countries' nationally representative diet and nutrition survey. [1,2] For example, between 1986-87 the UK population had especially low wholegrain intakes, at 2.5 serving per week, of which bread accounted for 48%. [2] Whereas, in 2002, 79% of Norwegian women consumed at least one to six serving of high fibre bread per week. [1] The distribution of food intakes by quantity may be distinct for other items too. For instance, Norwegian women consumed an average of 0.3 serving of fish per day [1], whereas British women consumed an average of 0.3 serving per week. [3]

© 2024 Friel C et al. *JAMA Network Open.*

eTable 1. Food items and factor loadings representing a 'healthy' prenatal dietary pattern

[a]MoBa		[a]ALSPAC	
**Food item**	**Factor loading**	**Food item**	**Factor loading**
Dried fruit	0.288	Fruit	0.51
Berries	0.192		
Traditional fruits	0.355		
Cooked vegetables	0.416	Greens	0.465
Leafy greens	0.356	Leafy greens	0.354
Raw vegetables	0.472	Salad	0.456
		Root vegetables	0.351
Nuts	0.191	Nuts	0.315
High fiber cereals	0.242	High fiber cereal	0.367
Wholemeal bread	0.327	[c]Wholemeal bread	-0.458
		[c]Granary bread	-0.352
[b]Other fish	0.189		
Oily fish	0.3	Oily fish	0.471
Lean fish	0.246	Lean fish	0.29
Soft drinks (sugar sweetened)	-0.394	Soft drinks	0.057
Soft drinks (artificial sweeteners)	-0.402		
Fries	-0.2	Fries	-0.409
White bread	-0.35	White bread	0.531
Processed meat	-0.416	Processed meat	-0.279
Meat (nonpoultry)	-0.228	Meat (nonpoultry)	-0.043

ALSPAC, Avon Longitudinal Study of Parents and Children; MoBa, The Norwegian Mother, Father, and Child Cohort Study

The food items selected have been matched across ALSPAC and MoBa where possible. [a]The proportion of variance explained by the factor is 10% in MoBa and 15% in ALSPAC. [b]Some categories were broken down to a greater extent in one dataset than the other, for example, fruit in MoBa had three broad categories but was one question in ALSPAC. [b]'Other fish' refers to fish in mixed dishes. To ensure the same food items were in each cohort we did not remove low factor loadings (<0.2). [c]In ALSPAC, negative factor loadings for wholemeal and granary bread could not be adjusted through rotation, and may reflect the low intakes in the general population of England around the early 1990s. [2]

© 2024 Friel C et al. *JAMA Network Open.*

eTable 2. Food items and factor loadings of the subgroups of a 'healthy' prenatal dietary pattern

MoBa		ALSPAC	
**Food item**	**Factor loading**	**Food item**	**Factor loading**
**Plant-based dietary pattern**			
Dried fruit	0.277	Fruit	0.427
Berries	0.263		
Traditional fruits	0.446		
Cooked vegetables	0.546	Greens	0.678
Leafy greens	0.516	Leafy greens	0.56
Raw vegetables	0.653	Salad	0.402
		Root vegetables	0.467
Nuts	0.129	Nuts	0.276
High fiber cereals	0.197	High fiber cereal	0.297
Wholemeal bread	0.149	Wholemeal bread	-0.318
		Granary bread	-0.247
**Fish-based dietary pattern**			
Oily fish	0.196	Oily fish	0.626
Lean fish	0.679	Lean fish	0.626
[a]Other fish	0.362		
**'Unhealthy' dietary pattern**			
Soft drinks (sugar sweetened)	0.911	Soft drinks	-0.107
Soft drinks (artificial sweeteners)	0.885		
Fries	0.185	Fries	0.59
White bread	0.167	White bread	-0.412
Processed meat	0.113	Processed meat	0.531
Meat (nonpoultry)	0.073	Meat (nonpoultry)	0.264

ALSPAC, Avon Longitudinal Study of Parents and Children; MoBa, The Norwegian Mother, Father, and Child Cohort Study
[a]'Other fish' refers to fish in mixed dishes.

© 2024 Friel C et al. *JAMA Network Open.*

eTable 3. Estimated nutrient intakes in ALSPAC and MoBa by level of adherence to a 'healthy' prenatal dietary pattern

	MoBa (Median (interquartile range))				ALSPAC (Median (interquartile range))			
**Nutrient**	Overall	Low	Medium	High	Overall	Low	Medium	High
**Total energy (kilocalories)**	2,229 (1,880, 2,653)	2,256 (1,891, 2,694)	2,163 (1,825, 2,557)	2,276 (1,930, 2,709)	1,701 (1,404, 2,008)	1,694 (1,365, 2,029)	1,694 (1,404, 2,003)	1,712 (1,434, 1,997)
**Carbohydrate (g)**	295 (244, 358)	297 (243, 365)	284 (235, 342)	305 (254, 367)	207 (171, 248)	208 (166, 252)	205 (170, 247)	208 (175, 245)
**Free sugars (g)**	52 (36, 76)	64 (42, 95)	49 (34, 69)	47 (33, 66)	53 (37, 75)	60 (40, 89)	53 (37, 74)	48 (34, 64)
**Fibre (g)**	76 (63, 93)	79 (65, 96)	74 (62, 91)	76 (62, 93)	69 (55, 85)	71 (56, 88)	69 (56, 85)	68 (54, 82)
**Total fat (g)**	29 (24, 36)	30 (25, 37)	28 (23, 35)	28 (23, 35)	28 (21, 36)	30 (23, 39)	28 (22, 36)	26 (20, 33)
**Saturated fat (g)**	24 (20, 30)	25 (20, 30)	23 (19, 29)	24 (20, 31)	23 (18, 28)	24 (18, 30)	23 (18, 28)	23 (18, 28)
**Monounsaturated fatty acid (mg)**	14 (11, 18)	14 (11, 19)	13 (10, 17)	13 (10, 17)	11 (9, 15)	10 (8, 14)	11 (9, 15)	12 (10, 16)
**Polyunsaturated fatty acid (g)**	84 (72, 99)	81 (69, 96)	83 (71, 96)	89 (76, 103)	68 (56, 81)	62 (50, 74)	68 (56, 81)	74 (62, 87)
**Protein (g)**	2,044 (1,457, 3,237)	1,562 (1,177, 2,139)	1,967 (1,474, 2,899)	3,136 (2,056, 4,699)	1,996 (1,655, 2,274)	1,804 (837, 2,020)	1,999 (1,773, 2,213)	2,215 (1,973, 2,850)
**Beta carotene (µg)**	261 (209, 326)	225 (181, 279)	252 (208, 307)	311 (256, 379)	238 (193, 287)	202 (162, 244)	236 (197, 280)	277 (235, 323)
**Folate (µg)**	18 (16, 21)	17 (15, 20)	18 (16, 21)	20 (17, 23)	15 (12, 19)	13 (10, 16)	15 (12, 18)	17 (14, 21)
**Niacin (mg)**	1,108 (773, 1,586)	971 (684, 1,411)	1,059 (754, 1,504)	1,303 (930, 1,808)	626 (488, 845)	573 (424, 745)	624 (495, 837)	685 (543, 984)
**Retinol equivalents (µg)**	2,229 (1,880, 2,653)	2,256 (1,891, 2,694)	2,163 (1,825, 2,557)	2,276 (1,930, 2,709)	1,701 (1,404, 2,008)	1,694 (1,365, 2,029)	1,694 (1,404, 2,003)	1,712 (1,434, 1,997)
**Riboflavin (mg)**	295 (244, 358)	297 (243, 365)	284 (235, 342)	305 (254, 367)	207 (171, 248)	208 (166, 252)	205 (170, 247)	208 (175, 245)
**Thiamin (mg)**	1.5 (1.2, 1.8)	1.4 (1.2, 1.7)	1.4 (1.2, 1.7)	1.6 (1.3, 1.9)	1.4 (1.1, 1.7)	1.2 (1.0, 1.5)	1.4 (1.2, 1.7)	1.6 (1.3, 1.8)
**Vitamin B6 (mg)**	1.5 (1.2, 1.8)	1.3 (1.1, 1.6)	1.4 (1.2, 1.7)	1.7 (1.5, 2.0)	1.9 (1.5, 2.2)	1.7 (1.4, 2.0)	1.8 (1.5, 2.2)	2.0 (1.7, 2.4)
**Vitamin B12 (µg)**	5.4 (4.1, 7.3)	5.1 (3.8, 7.0)	5.3 (4.0, 7.1)	5.8 (4.4, 7.7)	4.3 (3.1, 6.2)	3.6 (2.6, 4.8)	4.2 (3.1, 6.1)	5.6 (3.7, 7.1)

© 2024 Friel C et al. *JAMA Network Open.*

Nutrient	MoBa (Median (interquartile range))				ALSPAC (Median (interquartile range))			
	Overall	Low	Medium	High	Overall	Low	Medium	High
**Vitamin C (mg)**	146 (102, 206)	121 (81, 176)	137 (99, 189)	181 (134, 243)	74 (53, 100)	52 (39, 71)	74 (56, 95)	99 (77, 125)
**Vitamin D (μg)**	3.2 (2.1, 4.4)	3.0 (1.9, 4.1)	3.1 (2.1, 4.3)	3.5 (2.5, 4.8)	3.3 (2.3, 5.1)	2.7 (1.9, 3.6)	3.3 (2.4, 4.7)	4.7 (3.1, 6.3)
**Vitamin E (mg)**	10.0 (8.0, 12.8)	9.4 (7.4, 12.2)	9.6 (7.7, 12.0)	11.1 (9.0, 14.1)	7.7 (5.5, 10.8)	5.9 (4.3, 8.1)	7.9 (5.8, 10.8)	9.7 (7.2, 12.9)
**Calcium (mg)**	977 (753, 1,263)	936 (708, 1,234)	955 (738, 1,222)	1,039 (815, 1,322)	927 (745, 1,123)	875 (689, 1,084)	925 (741, 1,120)	978 (803, 1,160)
**Magnesium (mg)**	388 (321, 467)	355 (294, 427)	378 (317, 449)	433 (364, 514)	241 (195, 293)	203 (163, 244)	239 (199, 285)	283 (238, 333)
**Phosphorus (μg)**	1,640 (1,353, 1,984)	1,545 (1,270, 1,880)	1,605 (1,334, 1,923)	1,773 (1,484, 2,118)	1,234 (1,011, 1,470)	1,098 (888, 1,323)	1,231 (1,024, 1,457)	1,364 (1,159, 1,588)
**Potassium (mg)**	3,876 (3,225, 4,649)	3,515 (2,922, 4,218)	3,759 (3,180, 4,457)	4,370 (3,702, 5,155)	2,848 (2,387, 3,327)	2,668 (2,196, 3,171)	2,841 (2,395, 3,320)	3,007 (2,594, 3,466)
**Selenium (μg)**	53 (44, 62)	48 (40, 57)	52 (44, 61)	59 (50, 68)	67 (51, 85)	56 (43, 71)	66 (52, 83)	80 (63, 101)
**Sodium (mg)**	2,969 (2,516, 3,491)	2,985 (2,538, 3,505)	2,923 (2,479, 3,422)	3,004 (2,532, 3,546)	2,165 (1,750, 2,601)	2,069 (1,632, 2,526)	2,134 (1,748, 2,592)	2,273 (1,874, 2,685)
**Zinc (mg)**	11.0 (9.2, 13.0)	10.6 (8.9, 12.6)	10.8 (9.0, 12.7)	11.6 (9.7, 13.7)	8.0 (6.5, 9.7)	7.1 (5.7, 8.7)	8.0 (6.6, 9.6)	8.9 (7.4, 10.4)

ALSPAC, Avon Longitudinal Study of Parents and Children; MoBa, Norwegian Mother, Father, and Child Cohort Study
In MoBa, daily nutrient intakes were estimated using FoodCalc [4] and the Norwegian food composition database and excluded alcohol and nutritional supplements [5]. In ALSPAC, daily nutrient intakes were estimated based on average portion sizes [6] using the McCance and Widdowson's 5th edition food composition tables and excluded alcohol and nutritional supplements [7]. Iodine intakes from diet were unavailable in our version of MoBa data, however further research from MoBa has confirmed iodine status is low as conformed by urinary iodine concentration and was unrelated to a healthy diet [8]

© 2024 Friel C et al. *JAMA Network Open.*

eAppendix 2. Explanation of DAG

The aetiology of autism diagnosis and autism-associated traits is complex, and there are many potential confounders. We adjusted for a range of potential confounders most proximal to the mother, which likely have the strongest relation to maternal prenatal diet as well as autism diagnosis or autism-associated traits. However, to test this assumption we additionally adjusted for a range of paternal characteristics. Furthermore, children diagnosed with autism in later birthyears will be younger and may have more severe autism. If so, adjustment for birthyear could introduce bias and so we adjusted in the sensitivity analysis. This can help restrict the analyses to a minimum necessary adjustment set whilst providing an indication of the robustness of results to additional, albeit weaker, sources of potential confounding.

The DAG (eFigure 1) depicts potential confounders of the relationship between prenatal dietary pattern and autism diagnosis or autism-associated traits. Maternal age may cause maternal education, and both are potential causes of each downstream node (downstream to another variable), HPDP and autism diagnosis or autism-associated traits. Therefore, we adjusted for maternal education, maternal age, pre-pregnancy BMI, planned pregnancy, maternal depression, prenatal multivitamin use, prenatal alcohol intake, prenatal smoking, pre-pregnancy hypertension, and pre-pregnancy diabetes. The sensitivity analyses additionally adjusted for the following paternal characteristics; paternal education, paternal income (MoBa only), paternal BMI, paternal age, and birthyear. The ‘unmeasured/residual confounding’ recognises the limitations of our study design, in that we cannot be certain all potential confounders have been adjusted for.

eFigure 1. Directed Acyclic Graph (DAG) of relationship between 'healthy' prenatal dietary pattern and autism related outcomes.

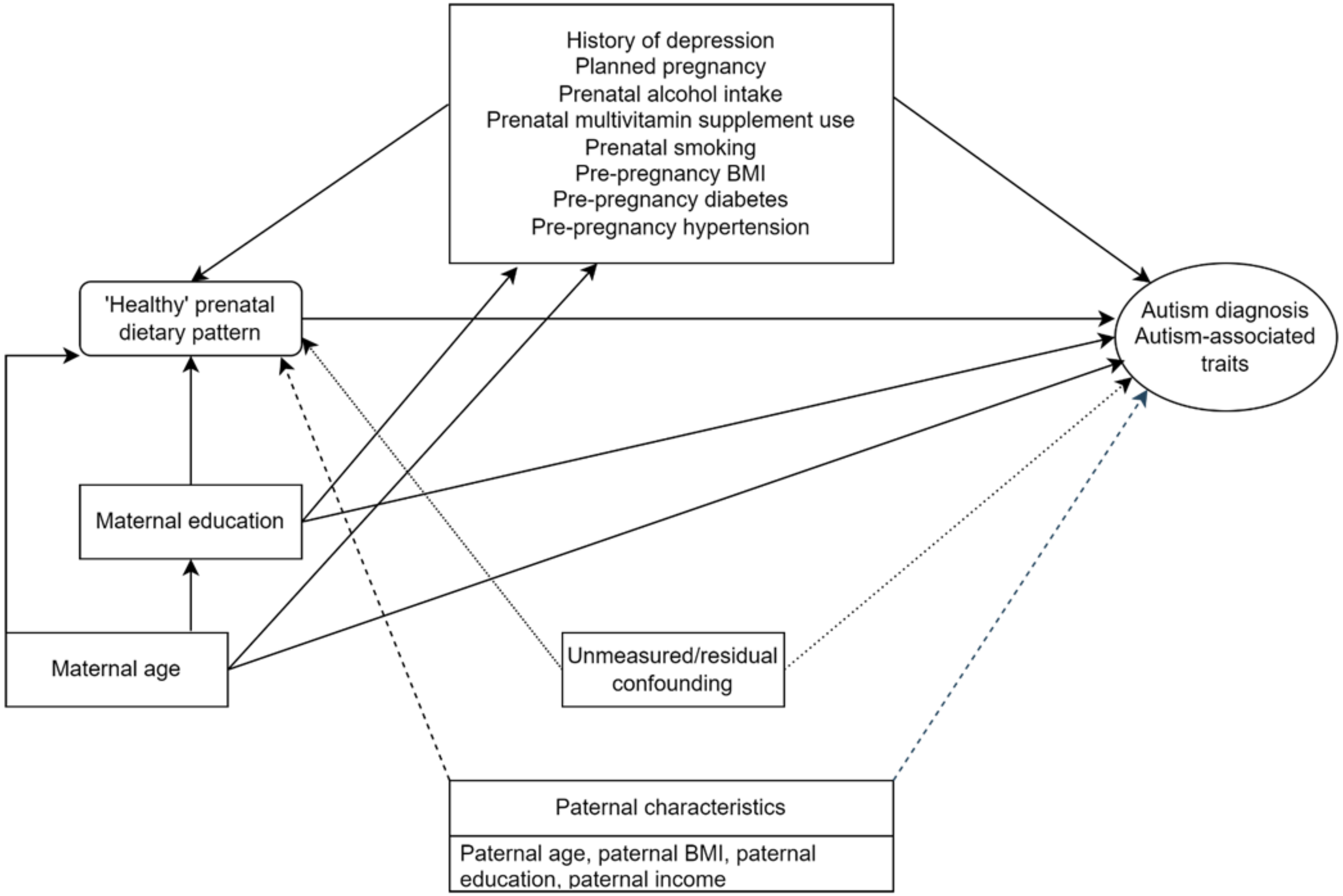

See text above, 'explanation of DAG. Solid line represents pathways between covariates included in the main analysis. Wide-dashed line indicates potential pathways with parental characteristics adjusted in sensitivity and close-dotted line indicates unmeasured and/or residual confounding.

© 2024 Friel C et al. *JAMA Network Open.*

eAppendix 3. Covariates

The Medical Birth Registry is a national health registry containing information about all births in Norway and was used in the Norwegian Mother, Father, and Child Cohort (MoBa) to obtain information on child sex, maternal age, and paternal age. All other covariates were obtained from the self-completed questionnaires. Maternal covariates from MoBa were: highest level of maternal education (9-year secondary school/ 1–2-year high school/ technical high school/ 3-year high school general studies, junior college/ regional technical college, 4-year university degree (Bachelor's degree, nurse, teacher, engineer)/ university, technical college more than 4 years (Master's degree, medical doctor, PhD)), maternal age (years), pre-pregnancy BMI (kg/m^2), pre-pregnancy depression (yes/no), history of anorexia nervosa, bulimia nervosa, or other eating disorder (yes/no) (referred to as eating disorder history (yes/no), history of anxiety (yes/no), planned pregnancy (yes/no), continuous use of multivitamin or folic acid supplement use from four weeks preconception to at least 12 weeks' gestation (yes/no) (referred to as prenatal multivitamin use), alcohol intake (yes/no), pre-pregnancy diabetes (yes/no), maternal smoking (yes/no), pre-pregnancy hypertension (yes/no), parity (nulliparous, one, two, three, four, five or more), cohabitation with partner (yes/no). Paternal covariates are: paternal BMI (kg/m^2), paternal age (years), paternal education (coded the same as maternal education), and paternal income (no income / under 150,000 Norwegian Krone (NOK) / 151,000-199,999 NOK / 200,000-299,999 NOK / 300,000-399,999 NOK / 400,000-499,999 NOK / over 500,000 NOK). Paternal covariates were all located from the maternal questionnaires as they were more complete.

Health records were used to obtain information on maternal diabetes, hypertension, and child sex. All other covariates were collected via self-completed questionnaires. Maternal covariates from ALSPAC were: maternal education (certificate of secondary education/none, vocational, ordinary level, advanced level, degree), maternal age (years), maternal pre-pregnancy BMI (kg/m^2), history of depression (yes/no), history of anorexia nervosa (yes/no) (referred to as history of eating disorder), history of other mental illness (yes/no), planned pregnancy (yes/no), any multivitamin or folic acid supplement use up to 18-weeks' gestation (yes/no), alcohol intake (yes/no), smoking (yes/no), pre-pregnancy diabetes (yes/no), and pre-pregnancy hypertension (yes/no), parity (nulliparous, one, two, three, four, five or more), anxiety or depression medication during pregnancy (yes/no), migraine medication (yes/no), sleep medication (yes/no). The paternal questionnaire was used to obtain paternal covariates on paternal age (years) and paternal BMI (kg/m^2), which were only available from the paternal questionnaire, but paternal education (certificate of secondary education/none, vocational, ordinary level, advanced level, degree) was obtained from the maternal questionnaire.

© 2024 Friel C et al. *JAMA Network Open.*

eAppendix 4. Sensitivity analyses

We recalculated the SCQ-3 and SCQ-8 scores based on fully complete responses to the SCQ without adjustment for phased speech. All SCQ scores were analysed as binary outcomes with a cut-off at the ≥93rd percentile to indicate a high level of autism-associated traits compared to a low level or no autism-associated traits. The overlap in children with high SCQ-3 score, high SCQ-8 score, and autism diagnosis are presented descriptively.

The precise composition of HPDP will vary across individuals and it is plausible that different facets of diet have a different association with the outcome. Therefore, we tested the consistency of our HPDP by deconstructing the HPDP into three food groups: plant-based, fish-based, and 'unhealthy'. We repeated the main analyses but exchanged HPDP for each food group and used the same covariates. The factor analyses were repeated using the same procedures as the main analyses but with different food items taken from the HPDP (see eTable 2 for list of food items). The categories were plant-based (included all fruit and vegetables, nuts, and granary/high fibre/wholemeal cereals and bread items in factor analyses), fish-based (included all fish items in factor analyses), and 'unhealthy' (included soft drinks, fries, white bread, processed meat, meat (nonpoultry) items in factor analyses). However, discrete dietary subgroups can be weaker measures [9] and so the interpretation focused on marked differences in the overall trend to avoid over interpretation.

Interactions were measured on a multiplicative scale in logistic regression models adjusted for the same covariates as the main analyses, using the Wald-test extended to imputed data using the D_2-statistic [10,11]. Interactions tested were between HPDP and: pre-pregnancy BMI (<25kg/m^2 / ≥25kg/m^2), child's sex (male / female), prenatal multivitamin supplement use (yes / no) and maternal education (<degree / ≥ degree).

We aimed to adjust for the minimally sufficient adjustment set in the main analysis and maintain comparability in analytic approach between MoBa and ALSPAC. To test the robustness of covariate selection, we adjusted for additional covariates in sensitivity analysis. All additional models were adjusted for the same covariates set as the main models (unless stated otherwise) which were: child sex, history of depression, maternal age, maternal education, planned pregnancy, prenatal alcohol intake, prenatal multivitamin supplement use, prenatal smoking, pre-pregnancy BMI, pre-pregnancy diabetes, pre-pregnancy hypertension. A second set of models additionally adjusted for the following paternal characteristics: BMI (kg/m^2) and age (years), education, and in MoBa only, paternal income, which was not available in ALSPAC during pregnancy. In a third set of models, we additionally adjusted for a range of covariates, most of which varied between MoBa and ALSPAC due to data availability. The additional covariates were: eating disorder history, parity, anxiety (MoBa only), cohabitation with partner (MoBa only), anxiety or depression medication (ALSPAC only), migraine medication (ALSPAC

only), sleep medication (ALSPAC only), other psychiatric history (ALSPAC only). Birthyear may represent cohort effects but can also reflect the child's age and so this was adjusted in a fourth set of models in sensitivity analysis. The fifth set of models repeated the adjustment set from the main analysis but omitted adjustment for prenatal multivitamin supplements.

Lastly, we repeated all main analysis based on complete cases in MoBa only, due to low counts in ALSPAC.

© 2024 Friel C et al. *JAMA Network Open.*

eTable 4. Variables used in imputation models.

MoBa, variables	Method	ALSPAC, variables	Method
autism diagnosis	nil missing	anxiety or depression medication	logreg
anxiety	logreg	birthweight	Predictive mean matching
birthweight	Predictive mean matching	child sex	nil missing
birthyear	polyreg	eating disorder history	logreg
child sex	logreg	gestational hypertension	logreg
cohabitation with partner	logreg	healthy prenatal dietary pattern	nil missing
eating disorder history	logreg	history of depression	logreg
healthy prenatal dietary pattern	nil missing	history of other mental illness	logreg
history of depression	logreg	hospitalisation before six years (child)	logreg
maternal age	nil missing	household income (child age 4 years)	polyreg
maternal education	polyreg	maternal age	Predictive mean matching
maternal income	polyreg	maternal education	polyreg
parity	polyreg	maternal social class	polyreg
paternal age	polyreg	migraine medication	logreg
paternal BMI	Predictive mean matching	parity	polyreg
paternal education	polyreg	paternal age	polyreg
paternal income	polyreg	paternal BMI	Predictive mean matching
planned pregnancy	logreg	planned pregnancy	logreg
prenatal alcohol intake	logreg	prenatal alcohol intake	logreg
prenatal multivitamin supplement use	logreg	prenatal multivitamin supplement use	logreg
prenatal smoking	logreg	prenatal smoking	logreg
pre-pregnancy BMI	Predictive mean matching	pre-pregnancy BMI	Predictive mean matching
pre-pregnancy diabetes	logreg	pre-pregnancy diabetes	logreg
pre-pregnancy hypertension	logreg	pre-pregnancy hypertension	logreg
SCQ-3	logreg	SCDC-8	logreg
SCQ-8	logreg	severe vomiting	logreg
total energy intake	nil missing	sleep medication	logreg
		total energy intake	nil missing
		townsend deprivation	polyreg

logreg, logistic regression, polyreg, Bayesian polytomous regression
ALSPAC, Avon Longitudinal Study of Parents and Children; BMI, body mass index; MoBa, The Norwegian Mother, Father, and Child Cohort Study; SCQ, social communication questionnaire; SCDC, social communication disorders checklist

© 2024 Friel C et al. *JAMA Network Open.*

eTable 5. Baseline characteristics of pregnancies with missing outcome data at age three in MoBa.

			Adherence to a 'healthy' prenatal dietary pattern (n (%))		
**Characteristic**	**Level**	**Overall**	**Low**	**Medium**	**High**
Number of pregnancies		32,986	11,287 (34.2)	10,801 (32.7)	10,898 (33.0)
Child sex	Male	16975 (51.5)	5888 (52.2)	5508 (51.0)	5579 (51.2)
	Female	15946 (48.3)	5371 (47.6)	5271 (48.8)	5304 (48.7)
	Missing	65 (0.2)	28 (0.2)	22 (0.2)	15 (0.1)
History of depression	No	29210 (88.6)	9913 (87.8)	9649 (89.3)	9648 (88.5)
	Yes	2790 (8.5)	1047 (9.3)	827 (7.7)	916 (8.4)
	Missing	986 (3.0)	327 (2.9)	325 (3.0)	334 (3.1)
Maternal age (years) (mean (SD))		29.9 (4.7)	28.8 (4.8)	30.1 (4.6)	31.0 (4.6)
	Missing	0	0	0	0
Maternal education	9-year elementary	1179 (3.6)	593 (5.3)	325 (3.0)	261 (2.4)
	1-2 years further education	1954 (5.9)	985 (8.7)	538 (5.0)	431 (4.0)
	Technical high school	4650 (14.1)	2116 (18.7)	1473 (13.6)	1061 (9.7)
	3-year high school general studies, junior college	4744 (14.4)	1917 (17.0)	1460 (13.5)	1367 (12.5)
	Regional technical college, 4-year university degree (Bachelor's degree, nurse, teacher, engineer)	11692 (35.4)	3684 (32.6)	4075 (37.7)	3933 (36.1)
	University, technical college, more than 4 years (Master's degree, medical doctor, PhD)	6461 (19.6)	1227 (10.9)	2204 (20.4)	3030 (27.8)
	Missing	2306 (7.0)	765 (6.8)	726 (6.7)	815 (7.5)
Planned pregnancy	No	6983 (21.2)	2638 (23.4)	2109 (19.5)	2236 (20.5)
	Yes	25122 (76.2)	8375 (74.2)	8429 (78.0)	8318 (76.3)
	Missing	881 (2.7)	274 (2.4)	263 (2.4)	344 (3.2)
Prenatal alcohol intake	No	24468 (74.2)	8617 (76.3)	8043 (74.5)	7808 (71.6)
	Yes	3215 (9.7)	977 (8.7)	1078 (10.0)	1160 (10.6)
	Missing	5303 (16.1)	1693 (15.0)	1680 (15.6)	1930 (17.7)
Prenatal multivitamin supplement use	No	22747 (69.0)	8388 (74.3)	7329 (67.9)	7030 (64.5)
	Yes	8055 (24.4)	2149 (19.0)	2776 (25.7)	3130 (28.7)
	Missing	2184 (6.6)	750 (6.6)	696 (6.4)	738 (6.8)
Prenatal smoking	No	26987 (81.8)	8573 (76.0)	8942 (82.8)	9472 (86.9)
	Yes	3089 (9.4)	1671 (14.8)	864 (8.0)	554 (5.1)
	Missing	2910 (8.8)	1043 (9.2)	995 (9.2)	872 (8.0)
Pre-pregnancy BMI kg/m^2 (mean (SD))		24.2 (4.5)	24.7 (4.8)	24.3 (4.4)	23.7 (4.2)
	Missing	1504 (4.6)	501 (4.4)	497 (4.6)	506 (4.6)

© 2024 Friel C et al. *JAMA Network Open.*

Characteristic	Level	Overall	Adherence to a 'healthy' prenatal dietary pattern (n (%))		
			Low	Medium	High
Pre-pregnancy diabetes	No	31833 (96.5)	10919 (96.7)	10414 (96.4)	10500 (96.3)
	Yes	167 (0.5)	41 (0.4)	62 (0.6)	64 (0.6)
	Missing	986 (3.0)	327 (2.9)	325 (3.0)	334 (3.1)
Pre-pregnancy hypertension	No	31805 (96.4)	10908 (96.6)	10440 (96.7)	10457 (96.0)
	Yes	356 (1.1)	125 (1.1)	112 (1.0)	119 (1.1)
	Missing	825 (2.5)	254 (2.3)	249 (2.3)	322 (3.0)
Autism diagnosis	No	32608 (98.9)	11107 (98.4)	10705 (99.1)	10796 (99.1)
	Yes	378 (1.1)	180 (1.6)	96 (0.9)	102 (0.9)
[a]SCQ-8	No	5861 (17.8)	1832 (16.2)	1945 (18.0)	2084 (19.1)
	Yes	643 (1.9)	231 (2.0)	201 (1.9)	211 (1.9)
	Missing	26482 (80.3)	9224 (81.7)	8655 (80.1)	8603 (78.9)
[a]SCQ-8-RR	No	5487 (16.6)	1734 (15.4)	1807 (16.7)	1946 (17.9)
	Yes	1017 (3.1)	329 (2.9)	339 (3.1)	349 (3.2)
	Missing	26482 (80.3)	9224 (81.7)	8655 (80.1)	8603 (78.9)
[a]SCQ-8-COM	No	5950 (18.0)	1861 (16.5)	1971 (18.2)	2118 (19.4)
	Yes	554 (1.7)	202 (1.8)	175 (1.6)	177 (1.6)
	Missing	26482 (80.3)	9224 (81.7)	8655 (80.1)	8603 (78.9)

BMI, body mass index; MoBa, Norwegian Mother, Father, and Child Cohort; SD, standard deviation; SCQ-RRB, restrictive and repetitive behaviours subdomain of SCQ; SCQ-SOC, communication skills subdomain of SCQ. The number following each outcome denotes the approximate age of the child in years when the measure was obtained. i.e. SCQ-SOC-8; social communication subdomain measured at age 8 years. [a]The presence of the outcome from each questionnaire was indicated by a high score.

© 2024 Friel C et al. *JAMA Network Open.*

eTable 6. Baseline characteristics of pregnancies with missing outcome data at age eight in MoBa.

Characteristic	Level	Overall	Adherence to a 'healthy' prenatal dietary pattern (n (%))		
			Low	Medium	High
Number of pregnancies		44,294	15,310 (34.6)	14,538 (32.8)	14,446 (32.6)
Child sex	Male	22774 (51.4)	7953 (51.9)	7426 (51.1)	7395 (51.2)
	Female	21455 (48.4)	7329 (47.9)	7090 (48.8)	7036 (48.7)
	Missing	65 (0.1)	28 (0.2)	22 (0.2)	15 (0.1)
History of depression	No	39462 (89.1)	13567 (88.6)	13022 (89.6)	12873 (89.1)
	Yes	3650 (8.2)	1349 (8.8)	1126 (7.7)	1175 (8.1)
	Missing	1182 (2.7)	394 (2.6)	390 (2.7)	398 (2.8)
Maternal age (years) (mean (SD))		29.9 (4.7)	28.7 (4.7)	30.0 (4.5)	30.9 (4.5)
	Missing	0	0	0	0
Maternal education	9-year elementary	1435 (3.2)	726 (4.7)	380 (2.6)	329 (2.3)
	1-2 years further education	2494 (5.6)	1257 (8.2)	698 (4.8)	539 (3.7)
	Technical high school	6234 (14.1)	2853 (18.6)	1986 (13.7)	1395 (9.7)
	3-year high school general studies, junior college	6445 (14.6)	2667 (17.4)	2001 (13.8)	1777 (12.3)
	Regional technical college, 4-year university degree (Bachelor’s degree, nurse, teacher, engineer)	15884 (35.9)	5081 (33.2)	5554 (38.2)	5249 (36.3)
	University, technical college, more than 4 years (Master’s degree, medical doctor, PhD)	8927 (20.2)	1755 (11.5)	3026 (20.8)	4146 (28.7)
	Missing	2875 (6.5)	971 (6.3)	893 (6.1)	1011 (7.0)
Planned pregnancy	No	8885 (20.1)	3355 (21.9)	2694 (18.5)	2836 (19.6)
	Yes	34370 (77.6)	11623 (75.9)	11542 (79.4)	11205 (77.6)
	Missing	1039 (2.3)	332 (2.2)	302 (2.1)	405 (2.8)
Prenatal alcohol intake	No	33232 (75.0)	11791 (77.0)	11001 (75.7)	10440 (72.3)
	Yes	4228 (9.5)	1279 (8.4)	1419 (9.8)	1530 (10.6)
	Missing	6834 (15.4)	2240 (14.6)	2118 (14.6)	2476 (17.1)
Prenatal multivitamin supplement use	No	30243 (68.3)	11360 (74.2)	9720 (66.9)	9163 (63.4)
	Yes	11383 (25.7)	3056 (20.0)	3968 (27.3)	4359 (30.2)
	Missing	2668 (6.0)	894 (5.8)	850 (5.8)	924 (6.4)
Prenatal smoking	No	36318 (82.0)	11660 (76.2)	12084 (83.1)	12574 (87.0)
	Yes	4026 (9.1)	2189 (14.3)	1131 (7.8)	706 (4.9)
	Missing	3950 (8.9)	1461 (9.5)	1323 (9.1)	1166 (8.1)
Pre-pregnancy BMI kg/m^2 (mean (SD))		24.2 (4.5)	24.7 (4.7)	24.3 (4.4)	23.7 (4.2)
	Missing	1828 (4.1)	628 (4.1)	579 (4.0)	621 (4.3)

© 2024 Friel C et al. *JAMA Network Open.*

Characteristic	Level	Overall	Adherence to a 'healthy' prenatal dietary pattern (n (%))		
			Low	Medium	High
Pre-pregnancy diabetes	No	42900 (96.9)	14857 (97.0)	14087 (96.9)	13956 (96.6)
	Yes	212 (0.5)	59 (0.4)	61 (0.4)	92 (0.6)
	Missing	1182 (2.7)	394 (2.6)	390 (2.7)	398 (2.8)
Pre-pregnancy hypertension	No	42857 (96.8)	14843 (96.9)	14088 (96.9)	13926 (96.4)
	Yes	465 (1.0)	159 (1.0)	162 (1.1)	144 (1.0)
	Missing	972 (2.2)	308 (2.0)	288 (2.0)	376 (2.6)
Autism diagnosis	No	43745 (98.8)	15061 (98.4)	14387 (99.0)	14297 (99.0)
	Yes	549 (1.2)	249 (1.6)	151 (1.0)	149 (1.0)
[a]SCQ-3	No	15754 (35.6)	5337 (34.9)	5254 (36.1)	5163 (35.7)
	Yes	2058 (4.6)	749 (4.9)	629 (4.3)	680 (4.7)
	Missing	26482 (59.8)	9224 (60.2)	8655 (59.5)	8603 (59.6)
[a]SCQ-3-RR	No	16056 (36.2)	5518 (36.0)	5333 (36.7)	5205 (36.0)
	Yes	1756 (4.0)	568 (3.7)	550 (3.8)	638 (4.4)
	Missing	26482 (59.8)	9224 (60.2)	8655 (59.5)	8603 (59.6)
[a]SCQ-3-COM (%)	No	16070 (36.3)	5376 (35.1)	5325 (36.6)	5369 (37.2)
	Yes	1742 (3.9)	710 (4.6)	558 (3.8)	474 (3.3)
	Missing	26482 (59.8)	9224 (60.2)	8655 (59.5)	8603 (59.6)

BMI, body mass index; MoBa, Norwegian Mother, Father, and Child Cohort; SD, standard deviation; SCQ-RRB, restrictive and repetitive behaviours subdomain of SCQ; SCQ-SOC, communication skills subdomain of SCQ. The number following each outcome denotes the approximate age of the child in years when the measure was obtained. i.e. SCQ-SOC-8; social communication subdomain measured at age 8 years. [a]The presence of the outcome from each questionnaire was indicated by a high score.

© 2024 Friel C et al. *JAMA Network Open.*

eTable 7. Baseline characteristics of pregnancies with missing outcome data at age eight in ALSPAC.

			Adherence to a 'healthy' prenatal dietary pattern (n (%))		
**Characteristic**	**Level**	**Overall**	**Low**	**Medium**	**High**
Number of pregnancies		4,481	1,845 (41.2)	1,486 (33.2)	1,150 (25.7)
History of depression	No	3604 (80.4)	1414 (76.6)	1217 (81.9)	973 (84.6)
	Yes	444 (9.9)	211 (11.4)	135 (9.1)	98 (8.5)
	Missing	433 (9.7)	220 (11.9)	134 (9.0)	79 (6.9)
Maternal age (years) (mean (SD))		26.7 (5.1)	25.06 (4.74)	26.96 (4.94)	28.81 (4.93)
	Missing	68 (1.5)	31 (1.7)	21 (1.4)	15 (1.3)
Maternal education	Certificate of secondary education/none	1322 (29.5)	791 (42.9)	369 (24.8)	162 (14.1)
	Vocational	507 (11.3)	241 (13.1)	179 (12.0)	87 (7.6)
	Ordinary level	1501 (33.5)	574 (31.1)	558 (37.6)	369 (32.1)
	Advanced level	767 (17.1)	176 (9.5)	279 (18.8)	312 (27.1)
	Degree	338 (7.5)	32 (1.7)	91 (6.1)	215 (18.7)
	Missing	46 (1.0)	31 (1.7)	10 (0.7)	5 (0.4)
Planned pregnancy	No	2750 (61.4)	1067 (57.8)	927 (62.4)	756 (65.7)
	Yes	1526 (34.1)	672 (36.4)	495 (33.3)	359 (31.2)
	Missing	205 (4.6)	106 (5.7)	64 (4.3)	35 (3.0)
Prenatal alcohol intake	No	1739 (38.8)	774 (42.0)	576 (38.8)	389 (33.8)
	Yes	643 (14.3)	211 (11.4)	216 (14.5)	216 (18.8)
	Missing	2099 (46.8)	860 (46.6)	694 (46.7)	545 (47.4)
Prenatal multivitamin supplement use	No	3333 (74.4)	1431 (77.6)	1100 (74.0)	802 (69.7)
	Yes	915 (20.4)	288 (15.6)	319 (21.5)	308 (26.8)
	Missing	233 (5.2)	126 (6.8)	67 (4.5)	40 (3.5)
Prenatal smoking	No	2934 (65.5)	1002 (54.3)	1019 (68.6)	913 (79.4)
	Yes	1373 (30.6)	747 (40.5)	416 (28.0)	210 (18.3)
	Missing	174 (3.9)	96 (5.2)	51 (3.4)	27 (2.3)
Pre-pregnancy BMI kg/m^2 (mean (SD))		23.1 (4.1)	23.3 (4.49)	23.3 (4.01)	22.5 (3.42)
	Missing	549 (1.2)	204 (1.1)	190 (1.3)	155 (1.4)
Pre-pregnancy diabetes	Yes	Not disclosed[a]	≤ 10[a]	≤ 10[a]	≤ 10[a]
	No	3839 (85.7)	1516 (82.2)	1297 (87.3)	1026 (89.2)
	Missing	Not disclosed[a]	Not disclosed[a]	Not disclosed[a]	Not disclosed[a]
Pre-pregnancy hypertension	No	4438 (99.0)	1823 (98.8)	1474 (99.2)	1141 (99.2)
	Yes	≤ 10[a]	≤ 10[a]	≤ 10[a]	≤ 10[a]
	Missing	Not disclosed[a]	Not disclosed[a]	Not disclosed[a]	Not disclosed[a]

ALSPAC, Avon Longitudinal Study of Parents and Children; BMI, body mass index; SD, standard deviation. The number following each outcome denotes the approximate age of the child in years when the measure was obtained.[a]Exact values have been removed as low counts are potentially disclosive.

© 2024 Friel C et al. *JAMA Network Open.*

eTable 8. Descriptive statistics for additional sociodemographic characteristics used in sensitivity analyses

**MoBa (n (%)**					
**Characteristic**		**Overall**	**Low**	**Medium**	**High**
	Number of pregnancies	84,548	28,183	28,182	28,183
Interactions					
Child sex	Male	43277 (51.2)	14465 (51.3)	14443 (51.2)	14369 (51.0)
	Female	41206 (48.7)	13690 (48.6)	13717 (48.7)	13799 (49.0)
	Missing	65 (0.1)	28 (0.1)	22 (0.1)	15 (0.1)
Maternal education category	No	52509 (62.1)	14439 (51.2)	18248 (64.8)	19822 (70.3)
	Yes	27216 (32.2)	12127 (43.0)	8412 (29.8)	6677 (23.7)
	Missing	4823 (5.7)	1617 (5.7)	1522 (5.4)	1684 (6.0)
Pre-pregnancy BMI category kg/m^2	<25	56032 (66.3)	17430 (61.8)	18511 (65.7)	20091 (71.3)
	≥ 25	25708 (30.4)	9818 (34.8)	8769 (31.1)	7121 (25.3)
	Missing	2808 (3.3)	935 (3.3)	902 (3.2)	971 (3.4)
Prenatal multivitamin supplement use	No	55706 (65.9)	20282 (72.0)	18268 (64.8)	17156 (60.9)
	Yes	24632 (29.1)	6506 (23.1)	8551 (30.3)	9575 (34.0)
	Missing	4210 (5.0)	1395 (4.9)	1363 (4.8)	1452 (5.2)
Additional adjustment for Birthyear					
	2002	4364 (5.2)	2017 (7.2)	1377 (4.9)	970 (3.4)
	2003	11463 (13.6)	4934 (17.5)	3614 (12.8)	2915 (10.3)
	2004	12127 (14.3)	4914 (17.4)	3934 (14.0)	3279 (11.6)
	2005	13264 (15.7)	4757 (16.9)	4405 (15.6)	4102 (14.6)
	2006	15116 (17.9)	4591 (16.3)	5203 (18.5)	5322 (18.9)
	2007	13753 (16.3)	3608 (12.8)	4800 (17.0)	5345 (19.0)
	2008	11454 (13.5)	2758 (9.8)	3889 (13.8)	4807 (17.1)
	2009	2848 (3.4)	555 (2.0)	922 (3.3)	1371 (4.9)
	Missing	159 (0.2)	49 (0.2)	38 (0.1)	72 (0.3)
Additional covariates for adjustment					
Cohabitation with partner	No	3247 (3.8)	1205 (4.3)	959 (3.4)	1083 (3.8)
	Yes	79567 (94.1)	26388 (93.6)	26666 (94.6)	26513 (94.1)
	Missing	1734 (2.1)	590 (2.1)	557 (2.0)	587 (2.1)
Eating disorder history	No	80275 (94.9)	26969 (95.7)	26872 (95.4)	26434 (93.8)
	Yes	2539 (3.0)	624 (2.2)	753 (2.7)	1162 (4.1)
	Missing	1734 (2.1)	590 (2.1)	557 (2.0)	587 (2.1)
History of anxiety	No	79874 (94.5)	26542 (94.2)	26720 (94.8)	26612 (94.4)
	Yes	2940 (3.5)	1051 (3.7)	905 (3.2)	984 (3.5)

© 2024 Friel C et al. *JAMA Network Open.*

MoBa (n (%)					
**Characteristic**		**Overall**	**Low**	**Medium**	**High**
History of anxiety	Missing	1734 (2.1)	590 (2.1)	557 (2.0)	587 (2.1)
Parity	Zero (primiparous)	38462 (45.5)	12827 (45.5)	12718 (45.1)	12917 (45.8)
	One	29876 (35.3)	10212 (36.2)	10125 (35.9)	9539 (33.8)
	Two	12571 (14.9)	3996 (14.2)	4177 (14.8)	4398 (15.6)
	Three	2652 (3.1)	831 (2.9)	864 (3.1)	957 (3.4)
	Four or more	828 (1.0)	268 (1.0)	260 (0.9)	300 (1.1)
	Missing	159 (0.2)	49 (0.2)	38 (0.1)	72 (0.3)
Additional adjustment for paternal characteristics					
Paternal age	<25 years	3803 (4.5)	2003 (7.1)	1069 (3.8)	731 (2.6)
	25-34 years	51800 (61.3)	18354 (65.1)	17456 (61.9)	15990 (56.7)
	35-44 years	26291 (31.1)	7241 (25.7)	8861 (31.4)	10189 (36.2)
	45-54 years	2063 (2.4)	427 (1.5)	636 (2.3)	1000 (3.5)
	≥ 55 years	164 (0.2)	29 (0.1)	35 (0.1)	100 (0.4)
	Missing	427 (0.5)	129 (0.5)	125 (0.4)	173 (0.6)
Paternal BMI kg/m^2 (mean (SD))		25.8 (3.3)	26.1 (3.5)	25.9 (3.3)	25.5 (3.1)
	Missing	4289 (5.1)	1400 (5.0)	1367 (4.9)	1522 (5.4)
Paternal income	No income	712 (0.8)	245 (0.9)	235 (0.8)	232 (0.8)
	Less than 150,000 NOK	4302 (5.1)	1563 (5.5)	1310 (4.6)	1429 (5.1)
	150-199,999 NOK	3243 (3.8)	1283 (4.6)	993 (3.5)	967 (3.4)
	200-299,999 NOK	16862 (19.9)	6939 (24.6)	5414 (19.2)	4509 (16.0)
	300-399,999 NOK	26214 (31.0)	9084 (32.2)	8890 (31.5)	8240 (29.2)
	400-499,999 NOK	13593 (16.1)	3751 (13.3)	4831 (17.1)	5011 (17.8)
	over 500,000 NOK	13346 (15.8)	3022 (10.7)	4554 (16.2)	5770 (20.5)
	Missing	6276 (7.4)	2296 (8.1)	1955 (6.9)	2025 (7.2)
Paternal education	9-year elementary	3575 (4.2)	1640 (5.8)	1086 (3.9)	849 (3.0)
	1-2 years further education	4430 (5.2)	2096 (7.4)	1336 (4.7)	998 (3.5)
	Technical high school	19673 (23.3)	8300 (29.5)	6417 (22.8)	4956 (17.6)
	3-year high school general studies, junior college	9337 (11.0)	3505 (12.4)	3116 (11.1)	2716 (9.6)
	Regional technical college, 4-year university degree (Bachelor's degree, nurse, teacher, engineer)	21106 (25.0)	6172 (21.9)	7378 (26.2)	7556 (26.8)
	University, technical college, more than 4 years (Master's degree, medical doctor, PhD)	18376 (21.7)	3785 (13.4)	6263 (22.2)	8328 (29.5)
	Missing	8051 (9.5)	2685 (9.5)	2586 (9.2)	2780 (9.9)
	Number of pregnancies	11,760	3920	3920	3920

© 2024 Friel C et al. *JAMA Network Open.*

ALSPAC (n (%))					
**Characteristic**		**Overall**	**Low**	**Medium**	**High**
Interactions					
Child sex	Male	6034 (51.3)	2017 (51.5)	1998 (51.0)	2019 (51.5)
	Female	5725 (48.7)	1902 (48.5)	1922 (49.0)	1901 (48.5)
	Missing	1 (0.0)	1 (0.0)	0 (0.0)	0 (0.0)
Maternal education category	High	4148 (35.3)	617 (15.7)	1264 (32.2)	2267 (57.8)
	Low	7542 (64.1)	3265 (83.3)	2636 (67.2)	1641 (41.9)
	Missing	70 (0.6)	38 (1.0)	20 (0.5)	12 (0.3)
Pre-pregnancy BMI category kg/m^2	<25	8275 (70.4)	2501 (63.8)	2693 (68.7)	3081 (78.6)
	≥ 25	2142 (18.2)	835 (21.3)	777 (19.8)	530 (13.5)
	Missing	1343 (11.4)	584 (14.9)	450 (11.5)	309 (7.9)
Prenatal multivitamin supplement use	No	8877 (75.5)	3067 (78.2)	3009 (76.8)	2801 (71.5)
	Yes	2568 (21.8)	683 (17.4)	823 (21.0)	1062 (27.1)
	Missing	315 (2.7)	170 (4.3)	88 (2.2)	57 (1.5)
Additional covariates for adjustment					
Anxiety or depression medication	No	11495 (97.7)	3791 (96.7)	3848 (98.2)	3856 (98.4)
	Yes	153 (1.3)	75 (1.9)	31 (0.8)	47 (1.2)
	Missing	112 (1.0)	54 (1.4)	41 (1.0)	17 (0.4)
Eating disorder history	No	10995 (93.5)	3607 (92.0)	3697 (94.3)	3691 (94.2)
	Yes	224 (1.9)	45 (1.1)	55 (1.4)	124 (3.2)
	Missing	541 (4.6)	268 (6.8)	168 (4.3)	105 (2.7)
History of other mental illness	No	10968 (93.3)	3581 (91.4)	3662 (93.4)	3725 (95.0)
	Yes	251 (2.1)	71 (1.8)	90 (2.3)	90 (2.3)
	Missing	541 (4.6)	268 (6.8)	168 (4.3)	105 (2.7)
Migraine medication	No	10707 (91.0)	3494 (89.1)	3554 (90.7)	3659 (93.3)
	Yes	941 (8.0)	372 (9.5)	325 (8.3)	244 (6.2)
	Missing	112 (1.0)	54 (1.4)	41 (1.0)	17 (0.4)
Parity	Zero (nulliparous)	5111 (43.5)	1541 (39.3)	1690 (43.1)	1880 (48.0)
	One	4006 (34.1)	1375 (35.1)	1333 (34.0)	1298 (33.1)
	Two	1601 (13.6)	555 (14.2)	551 (14.1)	495 (12.6)
	Three or more	450 (3.8)	167 (4.3)	167 (4.3)	116 (3.0)
	Missing	170 (1.4)	82 (2.1)	50 (1.3)	38 (1.0)
Sleep medication	No	11271 (95.8)	3709 (94.6)	3764 (96.0)	3798 (96.9)
	Yes	377 (3.2)	157 (4.0)	115 (2.9)	105 (2.7)
	Missing	112 (1.0)	54 (1.4)	41 (1.0)	17 (0.4)

© 2024 Friel C et al. *JAMA Network Open.*

ALSPAC (n (%))		ALSPAC (n (%))	ALSPAC (n (%))	ALSPAC (n (%))	ALSPAC (n (%))
**Characteristic**		**Characteristic**	**Characteristic**	**Characteristic**	**Characteristic**
Additional adjustment for paternal characteristics					
Paternal age category	<25 years	827 (7.0)	447 (11.4)	267 (6.8)	113 (2.9)
	25-29 years	2526 (21.5)	934 (23.8)	872 (22.2)	720 (18.4)
	29-34 years	2559 (21.8)	607 (15.5)	889 (22.7)	1063 (27.1)
	35-39 years	1071 (9.1)	217 (5.5)	327 (8.3)	527 (13.4)
	≥ 40 years	426 (3.6)	74 (1.9)	147 (3.8)	205 (5.2)
	Missing	4351 (37.0)	1641 (41.9)	1418 (36.2)	1292 (33.0)
Paternal BMI kg/m^2 (mean (SD))		25.2 (3.3)	25.4 (3.7)	25.2 (3.3)	24.9 (2.9)
	Missing	4089 (34.8)	1576 (40.2)	1333 (34.0)	1180 (30.1)
Paternal education	Certificate of secondary education/none	2884 (24.5)	1445 (36.9)	926 (23.6)	513 (13.1)
	Vocational	958 (8.1)	381 (9.7)	362 (9.2)	215 (5.5)
	Ordinary level	2389 (20.3)	824 (21.0)	874 (22.3)	691 (17.6)
	Advanced level	2970 (25.3)	822 (21.0)	1041 (26.6)	1107 (28.2)
	Degree	2039 (17.3)	183 (4.7)	561 (14.3)	1295 (33.0)
	Missing	520 (4.4)	265 (6.8)	156 (4.0)	99 (2.5)

ALSPAC, Avon Longitudinal Study of Parents and Children; BMI, body mass index; NOK, Norwegian Krone, MoBa, Norwegian Mother, Father, and Child Cohort; SD, standard deviation

© 2024 Friel C et al. *JAMA Network Open.*

eTable 9. Number and proportion of children across each outcome in MoBa

Outcome		SCQ-3 (n (%)		SCQ-8 (n (%)		Autism diagnosis (n (%)	
		No	Yes	No	Yes	No	Yes
**Number of participants**		46154	5408	36786	3468	83606	942
**Autism**	No	45789 (99.2)	5206 (96.3)	36606 (99.5)	3253 (93.8)	NA	NA
	Yes	365 (0.8)	199 (3.7)	180 (0.5)	213 (6.1)	NA	NA
	Missing	0 (0.0)	3 (0.1)	0 (0.0)	2 (0.1)	NA	NA
**[a]SCQ-8**	No	28284 (61.3)	2641 (48.8)	NA	NA	36606 (43.8)	180 (19.1)
	Yes	2116 (4.6)	709 (13.1)	NA	NA	3253 (3.9)	213 (22.6)
	Missing	15754 (34.1)	2058 (38.1)	NA	NA	43739 (52.3)	549 (58.3)
**[a]SCQ-RRB-8**	No	26641 (57.7)	2109 (39.0)	32288 (87.8)	1949 (56.2)	34104 (40.8)	133 (14.1)
	Yes	3759 (8.1)	1241 (22.9)	4498 (12.2)	1519 (43.8)	5755 (6.9)	260 (27.6)
	Missing	15754 (34.1)	2058 (38.1)	0 (0.0)	0 (0.0)	43739 (52.3)	549 (58.3)
**[a]SCQ-SOC-8**	No	28390 (61.5)	2878 (53.2)	36179 (98.3)	1039 (30.0)	36964 (44.2)	254 (27.0)
	Yes	2010 (4.4)	472 (8.7)	607 (1.7)	2429 (70.0)	2895 (3.5)	139 (14.8)
	Missing	15754 (34.1)	2058 (38.1)	0 (0.0)	0 (0.0)	43739 (52.3)	549 (58.3)
**[a]SCQ-3**	No	NA	NA	28284 (76.9)	2116 (61.0)	45789 (54.8)	365 (38.7)
	Yes	NA	NA	2641 (7.2)	709 (20.4)	5206 (6.2)	199 (21.1)
	Missing	NA	NA	5861 (15.9)	643 (18.5)	32603 (39.0)	378 (40.1)
**[a]SCQ-RRB-3**	No	44876 (97.2)	2202 (40.7)	28583 (77.7)	2439 (70.3)	46584 (55.7)	491 (52.1)
	Yes	1278 (2.8)	3206 (59.3)	2342 (6.4)	386 (11.1)	4411 (5.3)	73 (7.7)
	Missing	0 (0.0)	0 (0.0)	5861 (15.9)	643 (18.5)	32603 (39.0)	378 (40.1)
**[a]SCQ-SOC-3**	No	43286 (93.8)	3195 (59.1)	28385 (77.2)	2026 (58.4)	46143 (55.2)	338 (35.9)
	Yes	2868 (6.2)	2213 (40.9)	2540 (6.9)	799 (23.0)	4852 (5.8)	226 (24.0)
	Missing	0 (0.0)	0 (0.0)	5861 (15.9)	643 (18.5)	32603 (39.0)	378 (40.1)

Norwegian Mother, Father, and Child Cohort; NA, not applicable; n, number of mother-child dyads; SCQ-RRB, restrictive and repetitive behaviours subdomain of SCQ; SCQ-SOC, communication skills subdomain of SCQ. The number following each outcome denotes the approximate age of the child in years when the measure was obtained. i.e. SCQ-SOC-8; social communication subdomain measured at age 8 years. [a]The presence of the outcome from each questionnaire was indicated by a high score.

© 2024 Friel C et al. *JAMA Network Open.*

eFigure 2. Covariate balance plot for adherence to a 'healthy' prenatal dietary pattern in ALSPAC

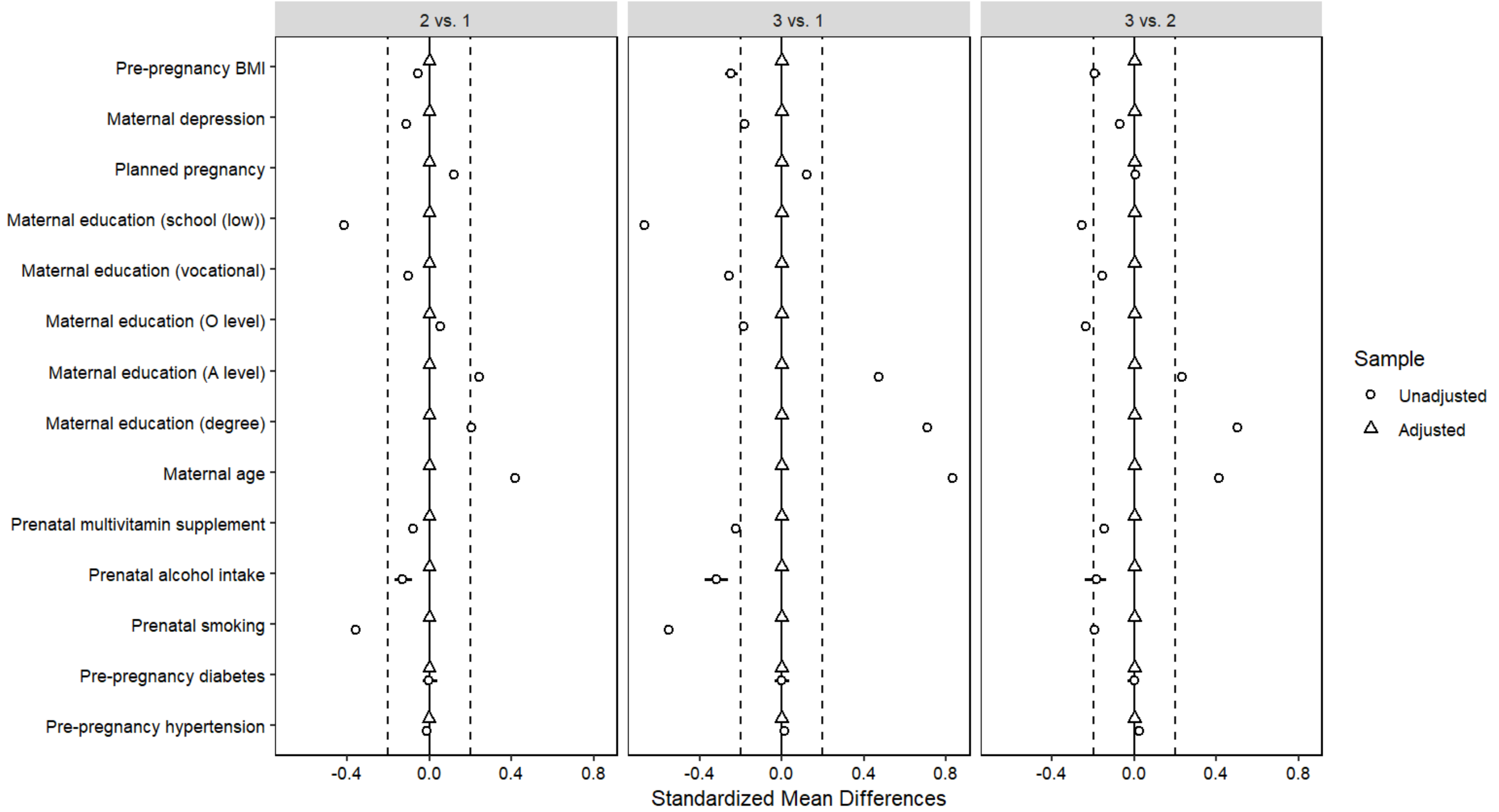

Good covariate balance was achieved. <0.1 standardised mean difference indicates a negligible difference between exposure groups. The numbers 1, 2, and 3 indicate low, medium, and high adherence to a 'healthy' prenatal dietary pattern, respectively. Similar covariate balance was achieved in all additional models used in sensitivity analysis and plots are available on request. Furthermore, we observed representation of each covariate across all levels of a HPDP (Table 1 and eTable 8). There were no extreme weights observed. Entropy balancing calibrates the weights which best balance the measured systematic and random error of the covariates against a set of base weights which best retain information. Propensity score estimation, entropy balancing combines covariate balancing constraints into the weighting process. This reduces extreme weights and avoids poor covariate balance [12]

© 2024 Friel C et al. *JAMA Network Open.*

eFigure 3. Covariate balance plot for adherence to a 'healthy' prenatal dietary pattern in MoBa

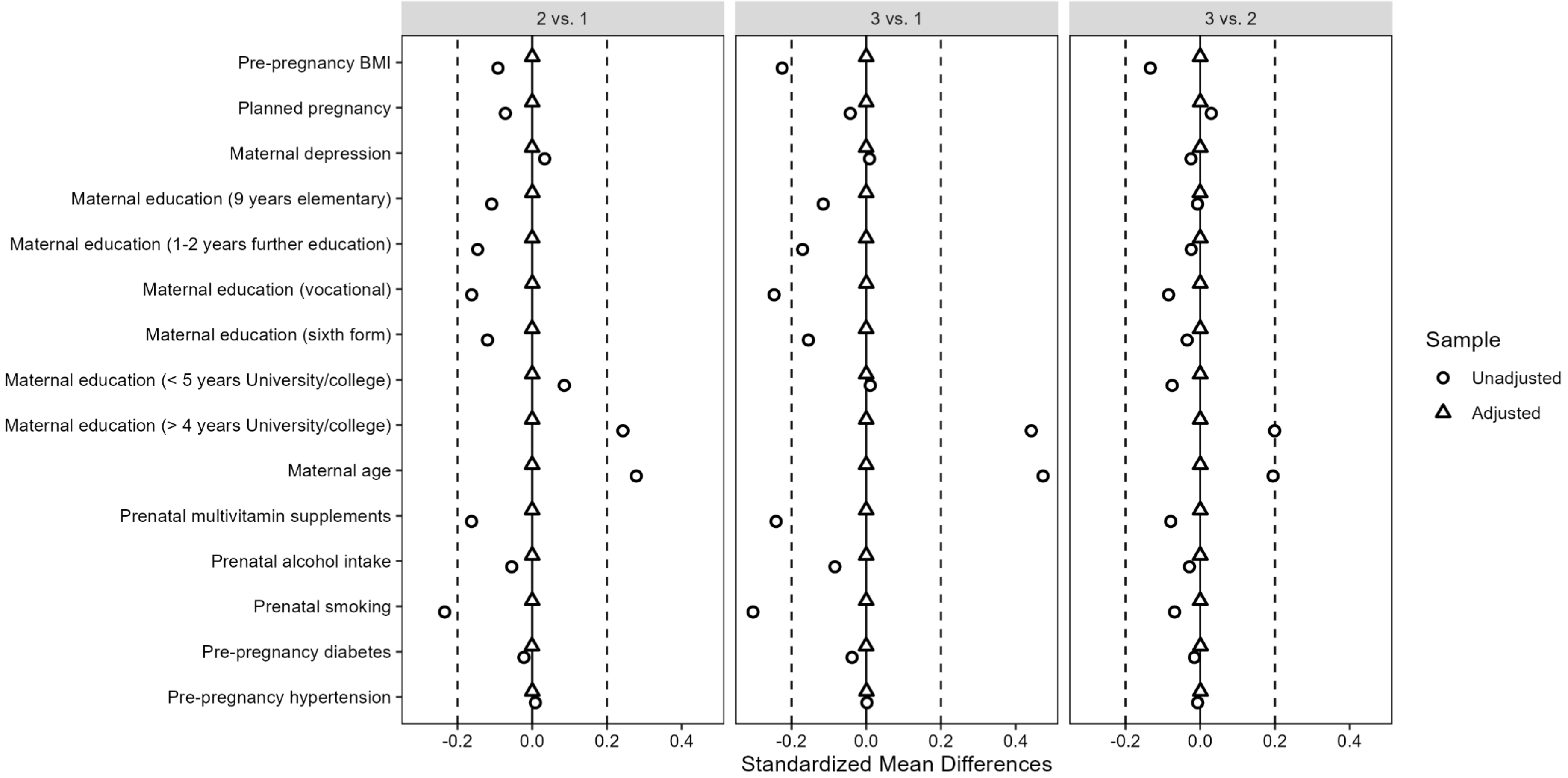

Good covariate balance was achieved. <0.1 standardised mean difference indicates a negligible difference between exposure groups and there were no extreme weights. The numbers 1, 2, and 3 indicate low, medium, and high adherence to a 'healthy' prenatal dietary pattern, respectively. Similar covariate balance was achieved in all additional models used in sensitivity analysis and plots are available on request. Furthermore, we observed representation of each covariate across all levels of a HPDP (Table 1 and eTable 8).

© 2024 Friel C et al. *JAMA Network Open.*

eTable 10. Unadjusted measures of association between a 'healthy' prenatal dietary pattern and autism diagnosis and autism-associated traits.

Outcome	[b]Adherence to 'healthy' prenatal dietary pattern	[b]OR (95% CI) unadjusted model
Autism diagnosis (MoBa)	Medium	0.71 (0.61, 0.83)
Autism diagnosis (MoBa)	High	0.68 (0.58, 0.80)
[a]SCQ-3 (MoBa)	Medium	0.85 (0.79, 0.91)
[a]SCQ-3 (MoBa)	High	0.86 (0.79, 0.93)
[a]SCQ-RRB-3 (MoBa)	Medium	0.92 (0.85, 0.99)
[a]SCQ-RRB-3 (MoBa)	High	1.05 (0.97, 1.14)
[a]SCQ-SOC-3 (MoBa)	Medium	0.80 (0.76, 0.85)
[a]SCQ-SOC-3 (MoBa)	High	0.68 (0.63, 0.73)
[a]SCQ-8 (MoBa)	Medium	0.86 (0.78, 0.94)
[a]SCQ-8 (MoBa)	High	0.80 (0.72, 0.88)
[a]SCQ-RRB-8 (MoBa)	Medium	0.86 (0.80, 0.94)
[a]SCQ-RRB-8 (MoBa)	High	0.87 (0.82, 0.93)
[a]SCQ-SOC-8 (MoBa)	Medium	0.88 (0.80, 0.96)
[a]SCQ-SOC-8 (MoBa)	High	0.79 (0.72, 0.88)
[a]SCDC-8 (ALSPAC)	Medium	0.68 (0.55, 0.84)
[a]SCDC-8 (ALSPAC)	High	0.59 (0.48, 0.74)

ALSPAC, Avon Longitudinal Study of Parents and Children; MoBa, Norwegian Mother, Father, and Child Cohort; CI, confidence intervals; OR, odds ratio; SCQ-RRB, restrictive and repetitive behaviours subdomain of SCQ; SCDC, social communication disorders checklist; SCQ-SOC, communication skills subdomain of SCQ. The number following each outcome denotes the approximate age of the child in years when the measure was obtained. i.e. SCQ-SOC-8; social communication subdomain measured at age 8 years. [a]The presence of the outcome for each questionnaire was indicated by a high score an represents a high level of autism-associated traits (measured binary (yes/no). Autism was measured binary (yes/no). [b]The reference category is low adherence to a 'healthy' prenatal dietary pattern.

© 2024 Friel C et al. *JAMA Network Open.*

eTable 11. Associations between a 'healthy' prenatal dietary pattern and autism diagnosis and autism-associated traits in MoBa and ALSPAC with a different adjustment set of covariates.

		[c]SCQ uncorrected	[d]Additional covariates	[e]Paternal characteristics	[f]Birthyear	[g]Unadjusted for prenatal multivitamin supplements
[a]Outcome	[b]Adherence to 'healthy' prenatal dietary pattern	OR (95% CI)	OR (95% CI)	OR (95% CI)	OR (95% CI)	OR (95% CI)
Autism diagnosis (MoBa)	Medium		0.77 (0.65, 0.90)	0.77 (0.65, 0.90)	0.80 (0.68, 0.95)	0.78 (0.66, 0.92)
Autism diagnosis (MoBa)	High		0.77 (0.65, 0.91)	0.77 (0.65, 0.91)	0.84 (0.71, 1.00)	0.79 (0.66, 0.93)
SCQ-3 (MoBa)	Medium	0.95 (0.85, 1.07)	1.08 (0.98, 1.18)	1.02 (0.94, 1.10)	1.01 (0.93, 1.09)	1.01 (0.93, 1.09)
SCQ-3 (MoBa)	High	1.08 (0.98, 1.20)	1.22 (1.12, 1.34)	1.05 (0.95, 1.16)	1.05 (0.96, 1.16)	1.05 (0.96, 1.15)
SCQ-RRB-3 (MoBa)	Medium	1.00 (0.89, 1.12)	1.13 (1.02, 1.24)	1.08 (1.01, 1.16)	1.07 (1.00, 1.15)	1.08 (1.01, 1.15)
SCQ-RRB-3 (MoBa)	High	1.37 (1.24, 1.51)	1.33 (1.20, 1.47)	1.26 (1.17, 1.36)	1.26 (1.16, 1.36)	1.26 (1.17, 1.36)
SCQ-SOC-3 (MoBa)	Medium	0.95 (0.85, 1.05)	0.92 (0.85, 0.99)	0.87 (0.82, 0.93)	0.88 (0.82, 0.93)	0.87 (0.82, 0.92)
SCQ-SOC-3 (MoBa)	High	1.07 (0.98, 1.17)	0.71 (0.66, 0.78)	0.76 (0.70, 0.82)	0.76 (0.70, 0.83)	0.76 (0.70, 0.82)
SCQ-8 (MoBa)	Medium	0.92 (0.83, 1.02)	0.93 (0.85, 1.02)	0.96 (0.88, 1.04)	0.95 (0.87, 1.04)	0.95 (0.87, 1.03)
SCQ-8 (MoBa)	High	0.96 (0.84, 1.09)	0.96 (0.86, 1.06)	0.96 (0.87, 1.05)	0.96 (0.88, 1.05)	0.95 (0.87, 1.04)
SCQ-RRB-8 (MoBa)	Medium	0.90 (0.80, 1.02)	0.91 (0.82, 1.01)	0.97 (0.90, 1.05)	0.96 (0.89, 1.04)	0.97 (0.90, 1.05)
SCQ-RRB-8 (MoBa)	High	1.07 (0.93, 1.24)	0.99 (0.89, 1.11)	1.01 (0.94, 1.08)	1.00 (0.92, 1.07)	1.01 (0.95, 1.08)
SCQ-SOC-8 (MoBa)	Medium	0.94 (0.78, 1.13)	0.97 (0.89, 1.05)	0.95 (0.87, 1.04)	0.95 (0.87, 1.05)	0.95 (0.87, 1.03)
SCQ-SOC-8 (MoBa)	High	0.98 (0.83, 1.17)	0.89 (0.81, 0.97)	0.91 (0.82, 1.01)	0.92 (0.83, 1.01)	0.90 (0.81, 1.00)
SCDC-8 (ALSPAC)	Medium		0.73 (0.59, 0.91)	0.75 (0.60, 0.94)		0.80 (0.63, 1.03)
SCDC-8 (ALSPAC)	High		0.73 (0.54, 0.99)	0.77 (0.57, 1.03)		0.81 (0.61, 1.06)

ALSPAC, Avon Longitudinal Study of Parents and Children; BMI, body mass index; MoBa, Norwegian Mother, Father, and Child Cohort; CI, confidence intervals; OR, odds ratio; SCQ-RRB, restrictive and repetitive behaviours subdomain of SCQ; SCDC, social communication disorders checklist; SCQ-SOC, communication skills subdomain of SCQ. The number following each outcome denotes the approximate age of the child in years when the measure was obtained. i.e. SCQ-SOC-8; social communication subdomain measured at age 8 years.

[a]The presence of the outcome from each questionnaire was indicated by a high score and represents a high level of autism-associated traits (measured binary (yes/no). Except autism which was binary (yes/no).

[b]The reference category is low adherence to a 'healthy' prenatal dietary pattern.

Model c adjusted for the same covariates as the main analysis which were: child sex, history of depression, maternal age, maternal education, planned pregnancy, prenatal alcohol intake, prenatal multivitamin supplement use, prenatal smoking, pre-pregnancy BMI, pre-pregnancy diabetes, pre-pregnancy hypertension. Model d adjusted for model c covariates and additionally adjusted for: eating disorder history, parity, anxiety (MoBa only), cohabitation (MoBa only), anxiety or depression medication (ALSPAC only), migraine medication (ALSPAC only), sleep medication (ALSPAC only), other psychiatric history (ALSPAC only). Model e adjusted for model c covariates and additionally adjusted for paternal age, paternal BMI, paternal education, paternal income (MoBa only). Model f adjusted for model c covariates and was additionally adjusted for birthyear. Model g adjusted for model c covariates but excluded prenatal multivitamin supplements.

© 2024 Friel C et al. *JAMA Network Open.*

eTable 12. Associations between a ‘healthy’ prenatal dietary pattern and food groups with autism diagnosis and autism-associated traits in ALSPAC and MoBa

		'Healthy' prenatal dietary pattern	[c]Plant-based food group	[c]Fish-based food group	[c]'Unhealthy' food group
**[a]Outcome**	**[b]Level of adherence**	**OR (95% CI)**	**OR (95% CI)**	**OR (95% CI)**	**OR (95% CI)**
Autism diagnosis (MoBa)	Medium	0.77 (0.66, 0.91)	0.94 (0.80, 1.10)	0.90 (0.77, 1.06)	0.89 (0.75, 1.05)
Autism diagnosis (MoBa)	High	0.78 (0.66, 0.92)	0.91 (0.77, 1.08)	0.98 (0.83, 1.14)	1.05 (0.89, 1.24)
SCQ-3 (MoBa)	Medium	1.02 (0.94, 1.10)	1.00 (0.94, 1.07)	0.95 (0.89, 1.01)	0.93 (0.86, 1.00)
SCQ-3 (MoBa)	High	1.06 (0.96, 1.16)	1.07 (1.00, 1.15)	0.94 (0.88, 1.01)	0.94 (0.88, 1.00)
SCQ-RRB-3 (MoBa)	Medium	1.08 (1.01, 1.16)	1.07 (0.99, 1.15)	0.95 (0.89, 1.02)	1.09 (1.00, 1.18)
SCQ-RRB-3 (MoBa)	High	1.27 (1.18, 1.37)	1.26 (1.15, 1.37)	0.94 (0.87, 1.01)	1.11 (1.03, 1.20)
SCQ-SOC-3 (MoBa)	Medium	0.87 (0.82, 0.93)	0.86 (0.80, 0.92)	0.92 (0.86, 0.98)	1.09 (1.00, 1.18)
SCQ-SOC-3 (MoBa)	High	0.76 (0.70, 0.82)	0.80 (0.74, 0.86)	0.95 (0.89, 1.02)	1.11 (1.03, 1.20)
SCQ-8 (MoBa)	Medium	0.96 (0.88, 1.04)	0.92 (0.86, 0.98)	0.93 (0.85, 1.01)	0.95 (0.87, 1.02)
SCQ-8 (MoBa)	High	0.96 (0.88, 1.05)	0.92 (0.86, 0.99)	0.95 (0.89, 1.02)	0.97 (0.89, 1.06)
SCQ-RRB-8 (MoBa)	Medium	0.97 (0.90, 1.05)	0.98 (0.92, 1.04)	0.94 (0.89, 1.00)	0.94 (0.89, 1.00)
SCQ-RRB-8 (MoBa)	High	1.01 (0.95, 1.08)	1.03 (0.97, 1.09)	0.93 (0.88, 0.99)	1.00 (0.95, 1.06)
SCQ-SOC-8 (MoBa)	Medium	0.96 (0.87, 1.04)	0.93 (0.86, 1.00)	0.96 (0.88, 1.05)	1.00 (0.91, 1.09)
SCQ-SOC-8 (MoBa)	High	0.91 (0.82, 1.01)	0.89 (0.82, 0.96)	0.98 (0.88, 1.08)	1.01 (0.93, 1.10)
SCDC-8 (ALSPAC)	Medium	0.73 (0.59, 0.91)	0.76 (0.62, 0.94)	0.87 (0.74, 1.04)	1.10 (0.89, 1.36)
SCDC-8 (ALSPAC)	High	0.74 (0.55, 0.98)	0.83 (0.66, 1.04)	0.87 (0.68, 1.10)	1.21 (0.99, 1.49)

CI, confidence intervals; OR, odds ratio; SCDC, social communication disorders checklist; SCQ-RRB, restrictive and repetitive behaviours domain of SCQ; SCDC, social communication disorders checklist; SCQ-SOC, communication skills domain of SCQ: SCQ, social communication questionnaire. The number following each outcome denotes the child’s age in years when the measure was obtained.

[a]Autism was measured binary (yes/no) and high SCQ and SCDC score indicates greater difficulties and represents a high level of autism-associated traits (measured binary (yes/no).

[b]The reference category is low adherence to a ‘healthy’ prenatal dietary pattern.

Adjusted covariates in each model; child sex, history of depression, maternal age, maternal education, planned pregnancy, prenatal alcohol intake, prenatal multivitamin supplement use, prenatal smoking, pre-pregnancy BMI, pre-pregnancy diabetes, pre-pregnancy hypertension. Each of the food groups (plant-based, fish-based, ‘unhealthy’) were simultaneous adjusted for each other.

[c]The correlation between each food group as a continuous score was below 0.50.

© 2024 Friel C et al. *JAMA Network Open.*

eTable 13. Associations between a 'healthy' prenatal dietary pattern with autism diagnosis and autism-associated traits in MoBa and ALSPAC: analyses are stratified by prenatal multivitamin supplement use, pre-pregnancy BMI, child sex and maternal education

	[b, c]Maternal education		[b, c] Prenatal supplement use		[b, c] Pre-pregnancy BMI		[b, c] Child sex	
[a]Outcome	Subgroup	OR (95% CI)	Subgroup	OR (95% CI)	Subgroup	OR (95% CI)	Subgroup	OR (95% CI)
**Autism diagnosis (MoBa)**	*P* = 0.93, D^2-statistic = 0.07		*P* = 0.83, D^2-statistic = 0.18		*P* = 0.47, D^2-statistic = 0.76		*P* = 0.11, D^2-statistic = 2.22	
Medium	< Degree	0.78 (0.59, 1.03)	Yes	0.74 (0.55, 0.99)	< 25 kg/m^2	0.78 (0.63, 0.96)	Male	0.73 (0.61, 0.87)
High	< Degree	0.77 (0.60, 1.00)	Yes	0.76 (0.57, 1.02)	< 25 kg/m^2	0.82 (0.66, 1.01)	Male	0.78 (0.65, 0.94)
Medium	≥ Degree	0.77 (0.60, 0.99)	No	0.80 (0.66, 0.96)	≥ 25 kg/m^2	0.78 (0.61, 1.00)	Female	0.99 (0.71, 1.40)
High	≥ Degree	0.77 (0.61, 0.97)	No	0.77 (0.63, 0.95)	≥ 25 kg/m^2	0.68 (0.51, 0.90)	Female	0.71 (0.48, 1.05)
**SCQ-3 (MoBa)**	*P* = 0.01, D^2-statistic = 4.80		*P* = 0.01, D^2-statistic = 4.60		*P* = 0.78, D^2-statistic = 0.25		*P* = 0.007, D^2-statistic = 5.47	
Medium	< Degree	0.95 (0.85, 1.05)	Yes	0.90 (0.78, 1.05)	< 25 kg/m^2	0.98 (0.90, 1.07)	Male	0.96 (0.87, 1.05)
High	< Degree	0.96 (0.85, 1.08)	Yes	1.14 (0.98, 1.33)	< 25 kg/m^2	1.07 (0.98, 1.16)	Male	0.96 (0.87, 1.06)
Medium	≥ Degree	1.04 (0.94, 1.16)	No	1.02 (0.94, 1.10)	≥ 25 kg/m^2	1.00 (0.89, 1.13)	Female	1.04 (0.91, 1.19)
High	≥ Degree	1.17 (1.03, 1.33)	No	1.03 (0.95, 1.12)	≥ 25 kg/m^2	1.05 (0.91, 1.22)	Female	1.26 (1.09, 1.46)
**SCQ-RRB-3 (MoBa)**	*P* = 0.51, D^2-statistic = 0.68		*P* = 0.13, D^2-statistic = 2.05		*P* = 0.52, D^2-statistic = 0.66		*P* = 0.20, D^2-statistic = 1.64	
Medium	< Degree	1.08 (0.94, 1.23)	Yes	0.98 (0.83, 1.15)	< 25 kg/m^2	1.07 (0.98, 1.17)	Male	1.06 (0.96, 1.17)
High	< Degree	1.26 (1.09, 1.44)	Yes	1.33 (1.16, 1.53)	< 25 kg/m^2	1.32 (1.20, 1.45)	Male	1.22 (1.12, 1.33)
Medium	≥ Degree	1.04 (0.94, 1.15)	No	1.08 (1.00, 1.18)	≥ 25 kg/m^2	1.04 (0.91, 1.18)	Female	1.06 (0.94, 1.19)
High	≥ Degree	1.32 (1.17, 1.48)	No	1.26 (1.16, 1.38)	≥ 25 kg/m^2	1.21 (1.06, 1.39)	Female	1.39 (1.21, 1.61)
**SCQ-SOC-3 (MoBa)**	*P* = 0.34, D^2-statistic = 1.09		*P* = 0.73, D^2-statistic = 0.32		*P* = 0.16, D^2-statistic = 1.85		*P* = 0.001, D^2-statistic = 7.86	
Medium	< Degree	0.81 (0.73, 0.90)	Yes	0.86 (0.75, 0.97)	< 25 kg/m^2	0.82 (0.77, 0.88)	Male	0.83 (0.77, 0.91)
High	< Degree	0.72 (0.63, 0.81)	Yes	0.77 (0.68, 0.89)	< 25 kg/m^2	0.73 (0.67, 0.80)	Male	0.67 (0.62, 0.73)
Medium	≥ Degree	0.90 (0.82, 0.99)	No	0.87 (0.81, 0.93)	≥ 25 kg/m^2	0.94 (0.84, 1.06)	Female	0.92 (0.83, 1.01)
High	≥ Degree	0.77 (0.70, 0.86)	No	0.74 (0.68, 0.81)	≥ 25 kg/m^2	0.77 (0.69, 0.87)	Female	0.90 (0.79, 1.02)
**SCQ-8 (MoBa)**	*P* = 0.006, D^2-statistic = 5.49		*P* = 0.93, D^2-statistic = 0.08		*P* = 0.25, D^2-statistic = 1.42		*P* = 0.05, D^2-statistic = 3.16	
Medium	< Degree	0.85 (0.74, 0.97)	Yes	0.96 (0.83, 1.12)	< 25 kg/m^2	1.00 (0.90, 1.11)	Male	0.90 (0.81, 0.99)
High	< Degree	0.95 (0.84, 1.08)	Yes	0.95 (0.80, 1.14)	< 25 kg/m^2	0.95 (0.85, 1.06)	Male	0.95 (0.85, 1.07)
Medium	≥ Degree	1.04 (0.94, 1.15)	No	0.95 (0.85, 1.06)	≥ 25 kg/m^2	0.87 (0.75, 1.01)	Female	1.07 (0.93, 1.24)
High	≥ Degree	0.91 (0.81, 1.01)	No	0.92 (0.83, 1.02)	≥ 25 kg/m^2	0.90 (0.79, 1.04)	Female	0.88 (0.72, 1.07)
**SCQ-RRB-8 (MoBa)**	*P* = 0.08, D^2-statistic = 2.75		*P* = 0.40, D^2-statistic = 0.94		*P* = 0.27, D^2-statistic = 1.36		*P* = 0.11, D^2-statistic = 2.26	
Medium	< Degree	0.90 (0.79, 1.01)	Yes	0.97 (0.86, 1.10)	< 25 kg/m^2	0.94 (0.87, 1.02)	Male	0.95 (0.85, 1.07)

© 2024 Friel C et al. *JAMA Network Open.*

[a]Outcome	[b, c] Maternal education		[b, c] Prenatal supplement use		[b, c] Pre-pregnancy BMI		[b, c] Child sex	
	Subgroup	OR (95% CI)	Subgroup	OR (95% CI)	Subgroup	OR (95% CI)	Subgroup	OR (95% CI)
High	< Degree	0.93 (0.81, 1.06)	Yes	1.04 (0.91, 1.19)	< 25 kg/m^2	1.03 (0.95, 1.12)	Male	0.95 (0.86, 1.04)
Medium	≥ Degree	1.00 (0.90, 1.12)	No	0.95 (0.86, 1.04)	≥ 25 kg/m^2	0.96 (0.83, 1.12)	Female	0.95 (0.86, 1.04)
High	≥ Degree	1.06 (0.96, 1.17)	No	0.98 (0.92, 1.06)	≥ 25 kg/m^2	0.93 (0.85, 1.03)	Female	1.08 (0.98, 1.20)
**SCQ-SOC-8 (MoBa)**	*P* = 0.27, D^2-statistic = 1.35		*P* = 0.34, D^2-statistic = 1.11		*P* = 0.024, D^2-statistic = 3.97		*P* = 0.02, D^2-statistic = 4.42	
Medium	< Degree	0.88 (0.77, 1.01)	Yes	1.03 (0.82, 1.29)	< 25 kg/m^2	1.03 (0.91, 1.16)	Male	0.90 (0.82, 0.99)
High	< Degree	0.88 (0.76, 1.02)	Yes	1.01 (0.81, 1.27)	< 25 kg/m^2	0.91 (0.79, 1.04)	Male	0.92 (0.82, 1.04)
Medium	≥ Degree	0.99 (0.88, 1.12)	No	0.93 (0.84, 1.03)	≥ 25 kg/m^2	0.81 (0.71, 0.92)	Female	1.04 (0.90, 1.21)
High	≥ Degree	0.88 (0.78, 0.99)	No	0.85 (0.76, 0.94)	≥ 25 kg/m^2	0.86 (0.73, 1.01)	Female	0.82 (0.70, 0.96)
**SCDC-8 (ALSPAC)**	*P* = 0.21, D^2-statistic = 1.57		*P* = 0.35, D^2-statistic = 1.07		*P* = 0.19, D^2-statistic = 1.69		*P* = 0.04, D^2-statistic = 3.36	
Medium	≥ A levels	0.57 (0.38, 0.87)	No	0.57 (0.38, 0.87)	< 25 kg/m^2	0.57 (0.38, 0.87)	Male	0.81 (0.61, 1.06)
High	≥ A levels	0.59 (0.36, 0.96)	No	0.59 (0.36, 0.96)	< 25 kg/m^2	0.59 (0.36, 0.96)	Male	0.89 (0.67, 1.19)
Medium	< A levels	0.86 (0.66, 1.12)	Yes	0.86 (0.66, 1.12)	≥ 25 kg/m^2	0.86 (0.66, 1.12)	Female	0.71 (0.48, 1.06)
High	< A levels	0.82 (0.62, 1.08)	Yes	0.82 (0.62, 1.08)	≥ 25 kg/m^2	0.82 (0.62, 1.08)	Female	0.48 (0.33, 0.70)

CI, confidence intervals; OR, odds ratio; P, p-value; SCDC, social communication disorders checklist; SCQ-RRB, restrictive and repetitive behaviours domain of SCQ; SCDC, social communication disorders checklist; SCQ-SOC, communication skills domain of SCQ: SCQ, social communication questionnaire. The number following each outcome denotes the child's age in years when the measure was obtained.

[a]Autism was measured binary (yes/no). High SCQ and SCDC score indicates greater difficulties and represents a high level of autism-associated traits (measured binary (yes/no).

[b]The reference category is low adherence to a 'healthy' prenatal dietary pattern.

[c]Adjusted covariates in each model; child sex, history of depression, maternal age, maternal education, planned pregnancy, prenatal alcohol intake, prenatal multivitamin supplement use, prenatal smoking, pre-pregnancy BMI, pre-pregnancy diabetes, pre-pregnancy hypertension.

© 2024 Friel C et al. *JAMA Network Open.*

eTable 14. Complete case analyses measuring the associations between adherence to a 'healthy' prenatal dietary pattern and autism diagnosis and autism-associated traits in MoBa.

[a]Outcome	Total sample (cases)	[b]Adherence to a healthy prenatal dietary pattern	[c]OR (95% CI)
Autism diagnosis (MoBa)	58553 (393)	Medium	0.80 (0.62, 1.02)
Autism diagnosis (MoBa)	58553 (393)	High	0.74 (0.57, 0.95)
High SCQ-3 score (MoBa)	32362 (2023)	Medium	1.03 (0.92, 1.16)
High SCQ-3 score (MoBa)	32362 (2023)	High	1.16 (1.03, 1.31)
High SCQ-RRB- 3 score (MoBa)	32341 (1590)	Medium	1.03 (0.90, 1.19)
High SCQ-RRB- 3 score (MoBa)	32341 (1590)	High	1.45 (1.26, 1.66)
High SCQ-SOC- 3 score (MoBa)	32296 (1924)	Medium	0.89 (0.80, 1.01)
High SCQ-SOC- 3 score (MoBa)	32296 (1924)	High	0.72 (0.63, 0.82)
High SCQ-8 score (MoBa)	26215 (1304)	Medium	1.03 (0.90, 1.19)
High SCQ-8 score (MoBa)	26215 (1304)	High	0.88 (0.76, 1.03)
High SCQ-RRB- 8 score (MoBa)	26215 (1833)	Medium	0.98 (0.82, 1.18)
High SCQ-RRB- 8 score (MoBa)	26215 (1833)	High	1.14 (0.95, 1.37)
High SCQ-SOC- 8 score (MoBa)	26215 (1054)	Medium	1.10 (0.94, 1.28)
High SCQ-SOC- 8 score (MoBa)	26215 (1054)	High	0.90 (0.76, 1.07)

CI, confidence intervals; OR, odds ratio; SCDC, social communication disorders checklist; SCQ-RRB, restrictive and repetitive behaviours domain of SCQ; SCDC, social communication disorders checklist; SCQ-SOC, communication skills domain of SCQ: SCQ, social communication questionnaire. The number following each outcome denotes the child's age in years when the measure was obtained.

[a]Autism diagnosis was measured binary (yes/no). High SCQ and SCDC score indicates greater difficulties and represents a high level of autism-associated traits (measured binary (yes/no).

[b]The reference category is low adherence to a 'healthy' prenatal dietary pattern.

[c]Adjusted covariates in each model; child sex, history of depression, maternal age, maternal education, planned pregnancy, prenatal alcohol intake, prenatal multivitamin supplement use, prenatal smoking, pre-pregnancy BMI, pre-pregnancy diabetes, pre-pregnancy hypertension.

© 2024 Friel C et al. *JAMA Network Open.*

eReferences

1. The NORBAGREEN 2002 study: Consumption of vegetables, potatoes, fruit, bread and fish in the Nordic and Baltic countries (NORDIC COUNCIL OF MINISTERS: FOOD) (2003).
2. Lang R, Thane CW, Bolton-Smith C, Jebb SA. Consumption of whole-grain foods by British adults: findings from further analysis of two national dietary surveys. Public Health Nutrition. 2003;6(5):479-484.
3. Whitton C, Nicholson SK, Roberts C, et al. National Diet and Nutrition Survey: UK food consumption and nutrient intakes from the first year of the rolling programme and comparisons with previous surveys. Br J Nutr. Dec 2011;106(12):1899-914.
4. Foodcalc v 1.3 diet, cancer and health project. Danish Cancer Society; 1998.
5. Norwegian Food Safety Authority. The Norwegian Food Composition Table Norwegian Food Safety Authority; 2005.
6. Ministry of Agriculture and Food. Food Portion Sizes. HMSO; 1991.
7. Rogers I, Emmett P, Team AS. Diet during pregnancy in a population of pregnant women in South West England. European Journal of Clinical Nutrition. 1998;52(4):246-250.
8. Brantsaeter AL, Garthus-Niegel S, Brandlistuen RE, Caspersen IH, Meltzer HM, Abel MH. Mild-to-moderate iodine deficiency and symptoms of emotional distress and depression in pregnancy and six months postpartum – Results from a large pregnancy cohort. Journal of Affective Disorders. 2022;318:347-356.
9. Hu FB. Dietary pattern analysis: a new direction in nutritional epidemiology. Current Opinion in Lipidology. 2002;13(1):3-9.
10. Allison PD. Missing data. Sage; 2002.
11. Grund S, Lüdtke O, Robitzsch A. Pooling ANOVA Results From Multiply Imputed Datasets. Methodology. 2016;12(3):75-88.
12. Hainmueller J. Entropy Balancing for Causal Effects: A Multivariate Reweighting Method to Produce Balanced Samples in Observational Studies. Political Analysis. 2012;20(1):25-46.

© 2024 Friel C et al. *JAMA Network Open.*
