# Supplementary material for: Healthy Prenatal Dietary Pattern and Offspring Autism
Source: JAMA Netw Open. 2024 Jul 18;7(7):e2422815. doi: 10.1001/jamanetworkopen.2024.22815 (PMC11258593; doi:10.1001/jamanetworkopen.2024.22815)
Supplement: Supplement 2. — Data Sharing Statement [file jamanetwopen-e2422815-s002.pdf]

Data Sharing Statement

Friel. Healthy Prenatal Dietary Pattern and Offspring Autism. *JAMA Netw Open*. Published July 17, 2024. doi:10.1001/jamanetworkopen.2024.22815

Data
Data available: No

Additional Information
Explanation for why data not available: Data from the Norwegian Mother, Father and Child Cohort Study and the Medical Birth Registry of Norway used in this study are managed by the national health register holders in Norway (Norwegian Institute of public health) and can be made available to researchers, provided approval from the Regional Committees for Medical and Health Research Ethics (REC), compliance with the EU General Data Protection Regulation (GDPR) and approval from the data owners. The consent given by the participants does not open for storage of data on an individual level in repositories or journals. Researchers who want access to data sets for replication should apply through helsedata.no. Access to data sets requires approval from The Regional Committee for Medical and Health Research Ethics in Norway and an agreement with MoBa. Furthermore, information on how to access the ALSPAC data is available on their website (www.bristol.ac.uk/alspac/researchers/access). Code for analyses are available on request.
